# Hyperpolarisation of weakly binding N-heterocycles using signal amplification by reversible exchange†

**DOI:** 10.1039/d0sc06907h

**Published:** 2021-03-23

**Authors:** Peter J. Rayner, Joseph P. Gillions, Valentin D. Hannibal, Richard O. John, Simon B. Duckett

**Affiliations:** Centre for Hyperpolarisation in Magnetic Resonance (CHyM), Department of Chemistry, University of York Heslington York YO10 5DD UK simon.duckett@york.ac.uk

## Abstract

Signal Amplification by Reversible Exchange (SABRE) is a catalytic method for improving the detection of molecules by magnetic resonance spectroscopy. It achieves this by simultaneously binding the target substrate (sub) and *para*-hydrogen to a metal centre. To date, sterically large substrates are relatively inaccessible to SABRE due to their weak binding leading to catalyst destabilisation. We overcome this problem here through a simple co-ligand strategy that allows the hyperpolarisation of a range of weakly binding and sterically encumbered N-heterocycles. The resulting ^1^H NMR signal size is increased by up to 1400 times relative to their more usual Boltzmann controlled levels at 400 MHz. Hence, a significant reduction in scan time is achieved. The SABRE catalyst in these systems takes the form [IrX(H)_2_(NHC)(sulfoxide)(sub)] where X = Cl, Br or I. These complexes are shown to undergo very rapid ligand exchange and lower temperatures dramatically improve the efficiency of these SABRE catalysts.

## Abstract 2

The scope of the hyperpolarisation method Signal Amplification by Reversible Exchange (SABRE) is dramatically expanded through the use of co-ligands to substrates that weakly interact with the active cataylst.

## Graphical abstract

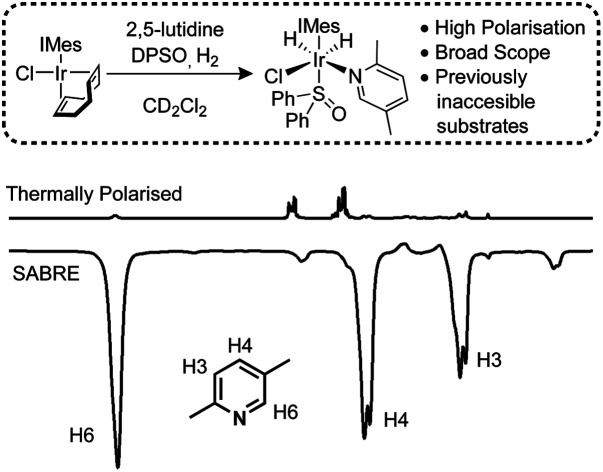

Hyperpolarised magnetic resonance is receiving increasing attention from both the analytical science and medical communities due to its ability to create signals that are many orders of magnitude higher than those normally detected under Boltzmann control.^1–6^ The time and cost benefits associated with this improvement have propelled this area of research forward over the past few decades. Two of the most prominent techniques used to create hyperpolarisation are dissolution Dynamic Nuclear Polarisation (d-DNP) and *Para*-Hydrogen Induced Polarisation (PHIP),^7,8^ which derive their non-Boltzmann spin energy level populations from interactions with unpaired electrons and *para*-hydrogen (*p*-H_2_, the singlet spin isomer of hydrogen), respectively. Both of these methods have been reviewed in detail.^3–5,9,10^

Signal Amplification by Reversible Exchange (SABRE) is a PHIP method that does not involve the chemical incorporation of *p*-H_2_ into the target substrate.^11,12^ Instead, under SABRE, spin order transfer proceeds catalytically through the temporary formation of a scalar coupling network between *p*-H_2_ derived hydride ligands and the substrate's nuclei whilst they are located in a transient metal complex. The most common catalysts are of the type [Ir(H)_2_(NHC)(sub)_3_]Cl (where NHC = N-heterocyclic carbene and sub = the substrate of interest, Fig. 1a),^13,14^ although other variants are known.^15–17^ For SABRE to be accomplished, the target substrate must be able to reversibly ligate to the metal centre and this limits the methods applicability; although several routes to overcome this have been reported.^18–20^ Recently, the use of bidentate ancillary ligands such as NHC-phenolates^16^ and phosphine-oxazoles^21^ has been shown to expand the applicability of SABRE for a variety of different ligands and solvents (Fig. 1b). For example, use of the PHOX ligand (PHOX = (2-diphenylphosphanyl)phenyl-4,5-dihydrooxazole) gives ^1^H NMR signal gains of up to 132-fold for 2-picoline; a substrate previously shown to be unpolarised under classic SABRE conditions.^22^

**Fig. 1:**
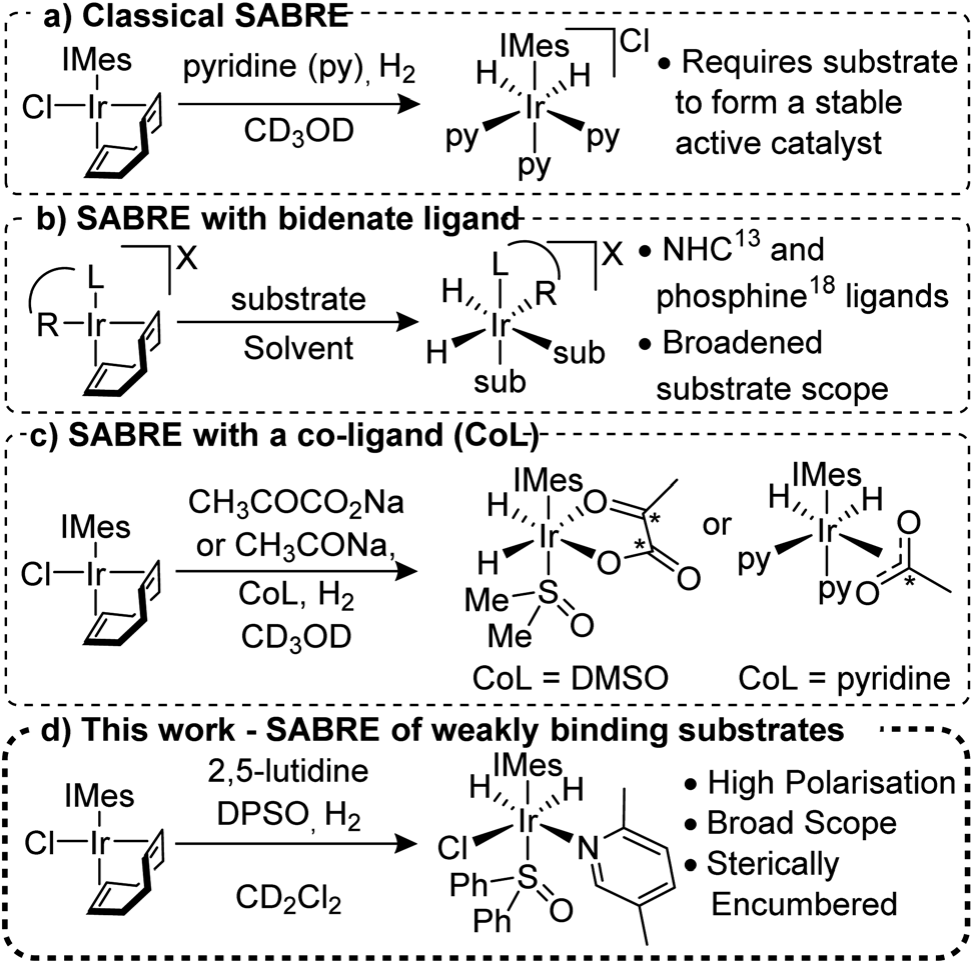
Development of the SABRE method for hyperpolarisation of a range of substrates.

The use of co-ligands to stabilise the active SABRE catalyst has proven successful for substrates that weakly associate to the catalyst (Fig. 1c). Of particular note is the hyperpolarisation of sodium [1,2]-^13^C_2_-pyruvate^23^ and sodium ^13^C-acetate^24^ which could be used as *in vivo* metabolic probes. The importance of co-ligands in breaking the chemical symmetry of the SABRE catalyst is also well established and co-ligands such as acetonitrile,^25^ sulfoxides,^23,26^ 1-methyl-1,2,3-triazole^27^ and substrate isotopologues^28^ have been employed.

We report here on the use of co-ligands to allow the NMR hyperpolarisation of weakly binding N-heterocyclic derived substrates with functionality in the *ortho*-position that have proven to be routinely inaccessible to the SABRE technique (Fig. 1d). ^1^H signal gains of up to 1442 ± 84-fold were obtained for some of these substituted pyridines at 9.4 T and the expansion of this approach to ^13^C and ^15^N detection and other N-heterocyclic motifs is also exemplified.

## Results and discussion

Under standard SABRE conditions, ([IrCl(COD)(IMes)] (5 mM) and substrate (20 mM) in either methanol-*d*_4_ or dichloromethane-*d*_2_ (0.6 mL)), we confirm^22^ that no ^1^H based polarisation enhancements are observed after polarisation transfer at 70 G under 3 bar *p*-H_2_ for an array of 2-substituted pyridines including 2-picoline and 2,5-lutidine. Additionally, there is no evidence of any hydride containing complexes being formed under these conditions when probed by either PHIP or low temperature NMR experiments. Presumably, this is due to the substrates weak ligation ability due to steric constraints around the metal centre caused by the substituents that lie *ortho* to the nitrogen binding site.

To overcome these limitations, we hypothesised that using a weakly binding co-ligand could allow for the formation of stable yet active SABRE catalysts. The key requirement for a co-ligand being that it will bind to the metal centre with comparable affinity to the target substrate, in order for the target substrate itself not to be completely displaced. Previously, sulfoxide co-ligands have fulfilled this role^23,26,29^ and, therefore, a sample was prepared that contained [IrCl(COD)(IMes)] (5 mM), 2,5-lutidine (20 mM) and DMSO-*d*_6_ (30 mM) in methanol-*d*_4_ and it was exposed to 3 bar H_2_ for 1 h at room temperature. After this time, *p*-H_2_ (3 bar) was added and the sample was shaken in a 70 G field before being rapidly inserted into a 9.4 T spectrometer for NMR analysis. Pleasingly, a 90 ± 7-fold ^1^H NMR signal enhancement was observed for the H6 resonance of 2,5-lutidine in the resulting spectrum when compared to the corresponding control spectrum obtained under Boltzmann conditions. The hydride region of this ^1^H NMR spectrum now contained hydride resonances for multiple (>6, see ESI†) PHIP enhanced dihydride complexes; each of which could be responsible for the observed SABRE transfer.

**Fig. 2:**
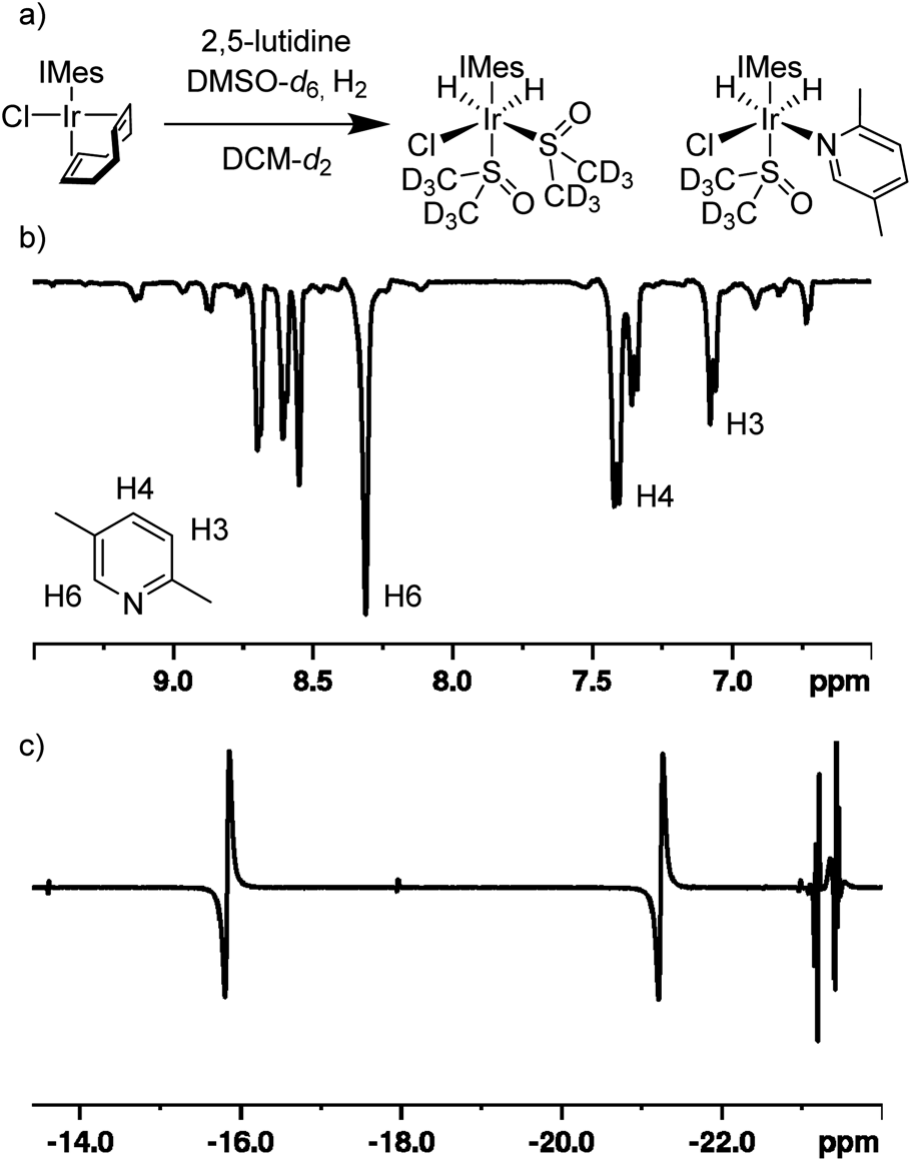
SABRE hyperpolarisation of 2,5-lutidine *via* precursor [IrCl(COD)(IMes)] and the co-ligand DMSO-*d*_6_ in dichloromethane-*d*_2_ solution. (a) Schematic of formation of complexes [IrCl(H)_2_(IMes)(DMSO-*d*_6_)_2_] and [IrCl(H)_2_(IMes)(DMSO-*d*_6_)(2,5-lutidine)]. (b) SABRE hyperpolarised ^1^H NMR spectrum of 2,5-lutidine after polarisation transfer at 70 G with 3 bar *p*-H_2_ and detection using a 90° flip angle. (c) PHIP enhanced hydride region of the hyperpolarised ^1^H NMR spectrum collected using a 45° flip angle.

When the same SABRE experiment was repeated using dichloromethane-*d*_2_ as the solvent, the observed signal enhancement for 2,5-lutidine significantly improves. Now, a 215 ± 13-fold signal gain was quantified for the H6 resonance of 2,5-lutidine. Additionally, just two hydride-containing complexes are now present in solution (Fig. 2). The first complex exhibits hydride resonances at *δ* −16.0 and −21.4 and is assigned to [IrCl(H)_2_(IMes)(DMSO-*d*_6_)_2_] on the basis of comparison to literature data.^29^ The second complex has hydride resonances at *δ* −23.0 and −23.5 and these are attributed to [IrCl(H)_2_(IMes)(DMSO-*d*_6_)(2,5-lutidine)]. In this species, the 2,5-lutidine and chloride ligands lie *cis* to one another and *trans* to hydride. These two complexes exist in a *ca*. 1 : 2 ratio respectively when the initial ratio of DMSO to 2,5-lutidine is 3 : 2. Under PHIP conditions, the enhanced hydride signals exhibit an intensity ratio of 1 : 4 after interrogation with a 45° pulse, thereby indicating a rapid H_2_ exchange pathway *via* [IrCl(H)_2_(IMes)(DMSO)(2,5-lutidine)]. In this spectrum, a minor hydride-containing complex also becomes visible with resonances at *δ* −13.57 and −17.96 which we attribute to the isomeric form of [IrCl(H)_2_(IMes)(DMSO-*d*_6_)(2,5-lutidine)] where the DMSO-*d*_6_ lies in the equatorial plane and the 2,5-lutidine is *trans* to the NHC ligand. Overall, rapid ligand exchange in this catalytic system is supported by the fact that the free H_2_ peak is broadened (30 Hz width at half height) and shifted to 4.52 ppm which differs from the usual 4.64 ppm value.

When the complex [IrCl(H)_2_(IMes)(DMSO-*d*_6_)_2_] is examined in the absence of 2,5-lutidine, the free H_2_ peak appears at 4.62 ppm with a half-width of 12 Hz which suggests a slower rate of hydride/hydrogen exchange in this complex when isolated. The rate of hydride ligand loss from [IrCl(H)_2_(IMes)(DMSO-*d*_6_)(2,5-lutidine)] to form H_2_ was also quantified to be 8.78 ± 0.40 s^−1^ at 273 K. The corresponding rate of 2,5-lutidine loss was 9.64 ± 0.21 s^−1^ at 273 K. Ligand exchange at 298 K was too rapid to be accurately determined using EXSY methods.

Density functional theory (DFT) confirms that [IrCl(H)_2_(IMes)(DMSO)(2,5-lutidine)] is the most energetically stable complex in solution, whilst [IrCl(H)_2_(IMes)(DMSO)_2_] is predicted to be 12.2 kJ mol^−1^ higher in energy. Other potential complexes such as [Ir(H)_2_(IMes)(DMSO)_2_(2,5-lutidine)]Cl or the classical SABRE complex [Ir(H)_2_(IMes)(2,5-lutidine)_3_]Cl are disfavoured with free energy values that are 71.4 and 74.7 kJ mol^−1^ higher than [IrCl(H)_2_(IMes)(DMSO)(2,5-lutidine)] respectively (see ESI†). Interestingly, we note that despite the fused bicyclic nature of quinoline, DFT suggests that [IrCl(H)_2_(IMes)(DMSO)(quinoline)] is 9.0 kJ mol^−1^ more stable than its 2,5-lutidine analogue; this ligand is hyperpolarised later in this manuscript.

### Role of sulfoxide co-ligand in active SABRE catalyst formation

The identity of the sulfoxide co-ligand proved to exhibit a significant impact on the level of the 2,5-lutidine NMR signal gains and the appearance of the free H_2_ signal. It was found that the use of methyl phenyl sulfoxide increased the ^1^H NMR signal gain for the H6 2,5-lutidine position to 319 ± 32 in an analogous dichloromethane-*d*_2_ sample. Even higher signal enhancements of 723 ± 38-fold were achieved when diphenylsulfoxide (DPSO) was the co-ligand. No evidence was observed for the formation of the analogous bis-sulfoxide complex, [IrCl(H)_2_(IMes)(DPSO)_2_], was seen by either PHIP or low temperature NMR measurements. [IrCl(H)_2_(IMes)(DPSO)(2,5-lutidine)], which has hydride resonances at *δ* −22.1 and −22.8, is the major product in this solution (see ESI† for full characterisation data). Now, the free H_2_ signal appears at 4.25 ppm with a peak half-width of 190 Hz, which indicates more rapid ligand exchange than in the corresponding DMSO derivative at 298 K.

As a consequence of changing the sulfoxide co-ligand from DMSO-*d*_6_ to DPSO, an increased concentration of the desired 2,5-lutidine containing SABRE catalyst results. Additionally, the rate of H_2_ exchange is increased. These factors result in a higher polarisation transfer level to 2,5-lutidine in the presence of DPSO than DMSO-*d*_6_. According to *ab initio* calculations, the favouring of [IrCl(H)_2_(IMes)(DPSO)(2,5-lutidine)] over [IrCl(H)_2_(IMes)(DPSO)_2_] has increased to 21.8 kJ mol^−1^; this is reflective of the cone angle of DPSO being 10% larger than that of DMSO.^30^ This increase in the steric parameter for the sulfoxide ligand may in turn reduce its binding affinity for the metal centre. Consequently, 2,5-lutidine may be able to more effectively compete for the equatorial ligand site.

Employing the aliphatic sulfoxides, tetramethylene sulfoxide and dibutylsulfoxide, resulted in signal gains for H6 of 2,5-lutidine of 131 ± 21 and 40 ± 8 fold respectively which are therefore similar to those achieved with DMSO-*d*_6_. Interestingly, no evidence of polarisation transfer into the undeuterated alkyl-sulfoxide co-ligands is observed in the ^1^H NMR spectra recorded after SABRE transfer at 70 G which may be due to short magnetic state lifetimes. In contrast, *ca*. 20-fold per proton signal gains were quantified for the CH resonances of the aryl rings in DPSO. This indicates that some polarisation transfer is occurring to the bound DPSO ligand. It is hypothesised therefore that deuteration of the sulfoxide co-ligand would further improve the efficiency of SABRE catalysis due to reduced spin-dilution.^28^ SABRE polarisation transfer to DMSO from [IrCl(H)_2_(DMSO)_2_(IMes)] has previously been reported.^29^

### Role of halide ligand in SABRE transfer

The effect of the halide ligand on the SABRE performance was explicitly probed by the synthesis of the related bromide and iodide containing complexes, prior to studying their reaction with 2,5-lutidine in the presence of DPSO and 3 bar *p*-H_2_. When [IrBr(COD)(IMes)] is utilised, the ^1^H NMR signal gain for the H6 proton of 2,5-lutidine was reduced to 570 ± 18 and a further reduction to a 171 ± 14-fold signal enhancement was quantified with the precatalyst [IrI(COD)(IMes)]. The reactivity of the corresponding dihydride products is readily apparent through their effects on the free H_2_ signal, which shifts to 4.19 and 4.04 ppm respectively with corresponding peak half-widths of 255 and 450 Hz. We deduce from these data that the rate of ligand exchange in [IrX(H)_2_(IMes)(DPSO)(2,5-lutidine)] increases for X = Cl < Br < I. Hence, these data suggest the signal enhancements become limited by catalyst lifetime.

### Effect of counter ion on active complex

Our study so far shows that neutral catalysts with a bound halide ligand are responsible for polarisation transfer to 2,5-lutidine in dichloromethane-*d*_2_. We wished to probe whether forcing the formation of a classical charged SABRE complex could further improve signal gains. To achieve this, we attempted to remove the halide ligand and replace it with a non-chelating counter ion. Therefore, the [Ir(COD)(IMes)(2,5-lutidine)]BF_4_ pre-catalyst was synthesised from [IrCl(COD)(IMes)], 2,5-lutidine and AgBF_4_ as detailed in the ESI.† Subsequently, this pre-catalyst was activated under the same conditions previously employed by exposure to 3 bar H_2_ in the presence of 2,5-lutidine (15 mM) and DPSO (20 mM) in dichloromethane-*d*_2_ for 1 h at room temperature. After SABRE transfer under 3 bar *p*-H_2_ in a 70 G polarisation transfer field a significantly diminished signal enhancement of just 19 ± 8 fold was detected for H6 of 2,5-lutidine. No hydride resonances were seen in these ^1^H NMR spectra and no discernible active SABRE complexes could be identified. However, the addition of excess Cl^−^ to this system reformed the expected [IrCl(H)_2_(IMes)(DPSO)(2,5-lutidine)] product and this restored the SABRE effect. SABRE transfer using [Ir(COD)(IMes)(2,5-lutidine)]BF_4_ was also attempted in methanol-*d*_4_, however, no polarisation transfer to 2,5-lutine was observed.

In a related experiment, using the [IrCl(COD)(IMes)] pre-catalyst in dichloromethane-*d*_2_ the addition of excess chloride resulted in no discernible change in SABRE signal enhancement which indicates that the polarisation transfer efficiency is independent of chloride concentration once the SABRE catalyst of type [IrCl(H)_2_(IMes)(DPSO)(2,5-lutidine)] is formed (see ESI†).

### Role of the NHC ligand in active SABRE transfer

The identity of the NHC has also been reported to influence the level of polarisation transfer resulting under SABRE.^14,31,32^ A series of six NHCs were therefore screened to probe their effect (a full summary is given in the ESI, Fig. S13†). Unlike in previous studies, deuteration of the IMes ligand resulted in no significant change in the ^1^H NMR signal enhancements seen for 2,5-lutidine.^15,28,33,34^ Introducing chlorine atoms into the imidazole backbone instead of protons resulted in a comparable 756 ± 74 fold signal gain. Additionally, when the methyl groups located in the *para*-position of the aryl rings were replaced with chlorine or –CO_2_Me, reduced signal enhancements of 663 ± 31 and 619 ± 44 were quantified respectively. However, when the *para*-methyl groups of IMes were replaced by *tert*-butyl, a 891 ± 49-fold signal enhancement for the H-6 resonance of 2,5-lutidine was quantified for DPSO as the co-ligand. This reflects a 30% improvement when compared to the results obtained with the IMes ligand.

The remaining ^1^H resonances of 2,5-lutidine also receive polarisation under these conditions with 635 ± 40, 868 ± 68, 240 ± 12 and 248 ± 8-fold signal gains for the H3, H4, *ortho*-CH_3_ and *meta*-CH_3_ sites respectively. When the SABRE sample containing the *tert*-butyl derived catalyst was cooled to 243 K, the presence of two minor hydride resonances at *δ* −13.5 and −18.7 is revealed. These are attributed to the analogous bis-DPSO complex that was described previously for the IMes derived catalyst. The ratio of the active SABRE catalyst to the bis-DPSO complex was estimated to be 98 : 2. Additionally, the rate of H_2_ exchange appears to be slower in the catalyst system formed when using the *tert*-butyl derived NHC catalyst. This is evidenced by the free H_2_ peak in the NMR spectra appearing at *δ*_H_ 4.36 with a peak half width of 70 Hz. This is a downfield shift when compared to that seen with [IrCl(H)_2_(IMes)(DPSO)(2,5-lutidine)], which exhibited a free H_2_ peak at *δ*_H_ 4.25 and a peak half width of 190 Hz. This indicates a reduction in the rate of H_2_ exchange which is consistent with an increase in catalyst lifetime; such observations would explain why higher signal enhancements are achieved if the complexes lifetimes are limiting.

### Effect of temperature on the SABRE hyperpolarisation of 2,5-lutidine

As stated, the broad free H_2_ and hydride signals are indicative of rapid ligand exchange on the active SABRE complexes at 298 K. Indeed, the rates of loss of 2,5-lutidine or hydride from the active catalyst were too fast to be accurately determined at 298 K using EXSY.^35^ It was hypothesised that cooling the SABRE samples may increase the lifetime of the catalyst to be closer to that which has been shown both computationally^36^ and experimentally^14^ to be optimal. Therefore, a sample containing the *tert*-butyl derived catalyst ([IrCl(COD)(1,3-bis(4-*tert*-butyl-2,6-dimethylphenyl)imidazole-2-ylidine)]), 2,5-lutidine and DPSO in dichloromethane-*d*_2_ was cooled to the desired temperature for 1 minute prior to the SABRE transfer being conducted in a 70 G field.

After cooling the sample to 283 K, a SABRE derived signal enhancement of 956 ± 38 was observed; a modest improvement on the 891 ± 49-fold signal gain previously detected at 298 K resulted. However, conducting the SABRE catalysis at 273 K yielded a significantly improved 1442 ± 84-fold signal gain at 9.4 T, which corresponds to a *ca*. 4.5% polarisation level. Further cooling the sample to 263 K did not give further benefits and a 934 ± 83-fold signal gain was quantified for 2,5-lutidine at this temperature. Therefore, we propose that conducting the SABRE hyperpolarisation of these weakly binding substrates at 273 K gives the optimum catalyst lifetime when using our co-ligand approach.

To probe this effect more precisely, the rate of 2,5-lutidine loss from the active catalyst was determined by EXSY. At 273 K the rate of this process was quantified to be 4.33 ± 0.02 s^−1^ which is extremely close to the *ca.* 4.5 s^−1^ which is predicted to be optimal by Barskiy *et al.*^36^ At both 283 and 263 K, these values deviate away from optimum and rates of 14.84 ± 0.50 and 0.89 ± 0.02 s^−1^ are quantified respectively. Eyring–Polanyi analysis across a temperature range of 243–283 K reveals a Δ*G*^≠298^ 59.89 kJ mol^−1^ for this process. When probing the loss of axially bound DPSO from [IrCl(H)_2_(IMes)(DPSO)(2,5-lutidine)] at 273 K, no exchange could be detected on the timescale of SABRE using EXSY.

### Expanding the substrate scope

**Fig. 3:**
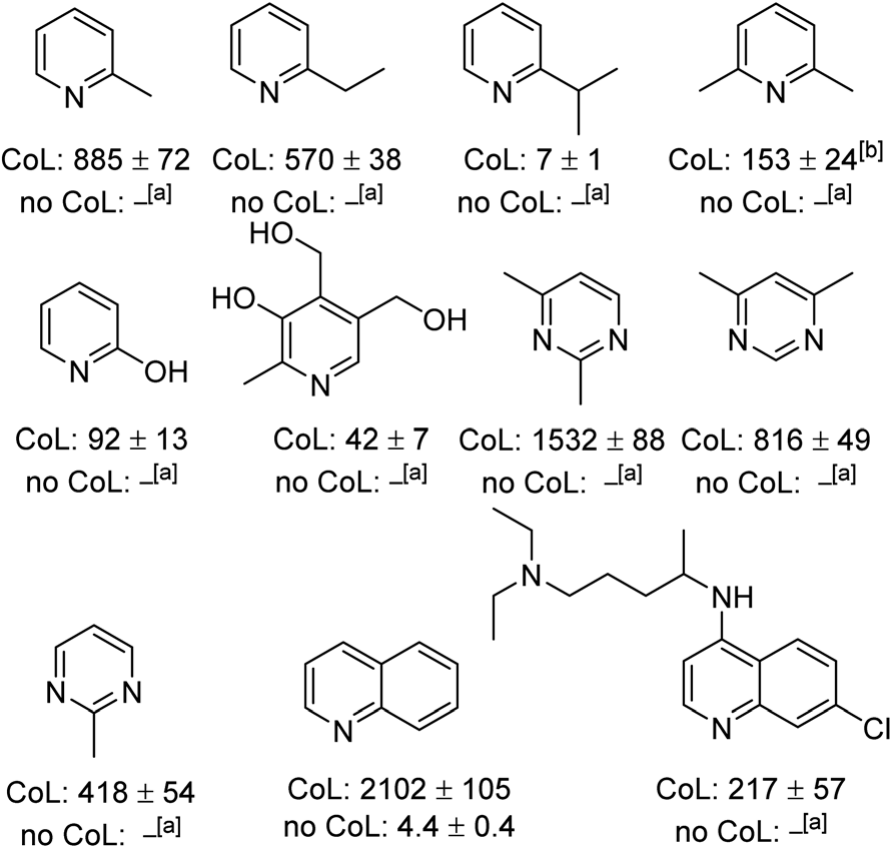
^1^H NMR SABRE hyperpolarisation levels (fold) for the indicated *ortho* proton achieved in a range of weakly binding N-heterocycles at 9.4 T under the following conditions: [IrCl(COD)(1,3-bis(4-*tert*-butyl-2,6-dimethylphenyl)imidazole-2-ylidine)] (5 mM), DPSO (20 mM) and substrate (20 mM) in dichloromethane-*d*_2_ with 3 bar *p*-H_2_ after polarisation transfer a 70 G field after cooling the sample for 1 minute at 273 K. CoL = signal enhancement for *ortho*-^1^H in the presence of DPSO. No CoL = signal enhancement for *ortho*-^1^H in the absence of DPSO. ^[a]^No observable signal enhancement. ^[b]^Signal for *meta* proton.

The generality of this method to sensitise the NMR detection of other weakly binding substrates with functional groups adjacent to their ligation site was also investigated. Fig. 3 shows the structures of the 11 other substrates examined in this study. They have been made amenable to SABRE by this co-ligand strategy. For clarity, their corresponding ^1^H NMR signal enhancements are displayed with and without the DPSO co-ligand. At 298 K, 2-picoline, which until recently^21^ has been previously reported as being unable to receive SABRE derived polarisation,^22^ yields a 403 ± 49-fold *ortho*-proton signal enhancement at 9.4 T when examined using our co-ligand approach. When repeating the SABRE transfer at 273 K, a 885 ± 72-fold signal enhancement is observed at 9.4 T which equates to *ca*. 1.25% polarisation. This compares favourably to use of the bidentate PHOX ligand;^21^ this yields ^1^H SABRE NMR signal gains of 132-fold for the *ortho*-proton of 2-picoline at 8.5 T (*ca*. 0.45% polarisation).

More sterically demanding 2-ethyl pyridine and 2-isopropylpyridine also become hyperpolarised by SABRE but their *ortho* proton signal gains are now just 83 ± 6 and 1.4 ± 0.2-fold respectively after SABRE transfer at 298 K. Interestingly, the decrease in signal enhancement is mirrored by a shift in the resonance of the free H_2_ and an increase in peak half-width. For 2-picoline, the free H_2_ resonance appears at 4.27 ppm with a peak half width of 150 Hz, whereas for 2-ethyl and 2-ispropyl pyridine the H_2_ resonance is seen at 3.36 and 2.27 ppm, with peak half widths of *ca*. 550 and 700 Hz respectively. This highlights how both ligand exchange and SABRE enhancements remain sensitive to ligand binding effects even in related systems despite this co-ligand strategy. Pleasingly, by conducting the SABRE catalysis at 273 K, the signal gains for 2-ethyl pyridine and 2-isopropyl pyridine could be also improved to 570 ± 38 and 7 ± 1-fold respectively.

Additionally, we were able to transfer SABRE derived hyperpolarisation to 2,6-lutidine and a 153 ± 24-fold signal gain for the *meta* proton was quantified after SABRE at 273 K. This sterically hindered and weakly binding substrate is reported to be unable to receive magnetisation under standard SABRE conditions^22^ or when using the bidentate PHOX catalyst.^21^ 2-Hydroxy pyridine also gave improved signal enhancements when DPSO was utilised. The signal enhancement for its H6 resonance was 92 ± 13-fold at 9.4 T. Similarly, the pyrimidine motif showed substantial signal improvements could be achieved in a range of mono and bis-methylated derivatives. A signal gain of 1532 ± 88-fold was quantified for 2,4-dimethylpyrimidine (*ca.* 4.8% polarisation) which is significant as no signal gain was recorded under standard SABRE conditions. 2-Methylpyrimidine also performs well using the optimised conditions and a 418 ± 54-fold signal enhancement is quantified. Very recently the hyperpolarisation of 2-picoline has been reported to be up to 25 ± 7-fold using an acetonitrile co-ligand in methanol-*d*_4_.^37^

The SABRE hyperpolarisation of quinoline is also significantly improved to 2102 ± 105-fold when compared to just 4.4 ± 0.4-fold when no DPSO is present in dichloromethane-*d*_2_. The SABRE hyperpolarisation of quinoline in methanol-*d*_4_ has previously been reported to be *ca.* 60-fold at 9.4 T.^38^ Finally, chloroquine, an anti-viral agent that has recently gained significant attention due to its use as a prospective COVID-19 treatment,^39–41^ also showed ^1^H SABRE polarisation. In the presence of DPSO, a 217 ± 57-fold signal gain was quantified, however, when it is absent no signal gain is observed. The appearance of the hydride region in these experiments is consistent with the formation of the corresponding highly reactive complex of type [IrCl(H)_2_(NHC)(DPSO)(substrate)] as the major product. The significant enhancements seen for quinoline are consistent with the DFT findings presented earlier which suggest greater binding affinity for quinoline than 2,5-lutidine despite the bicyclic nature of the ring system.

### SABRE polarisation transfer to ^13^C and ^15^N nuclei

**Fig. 4:**
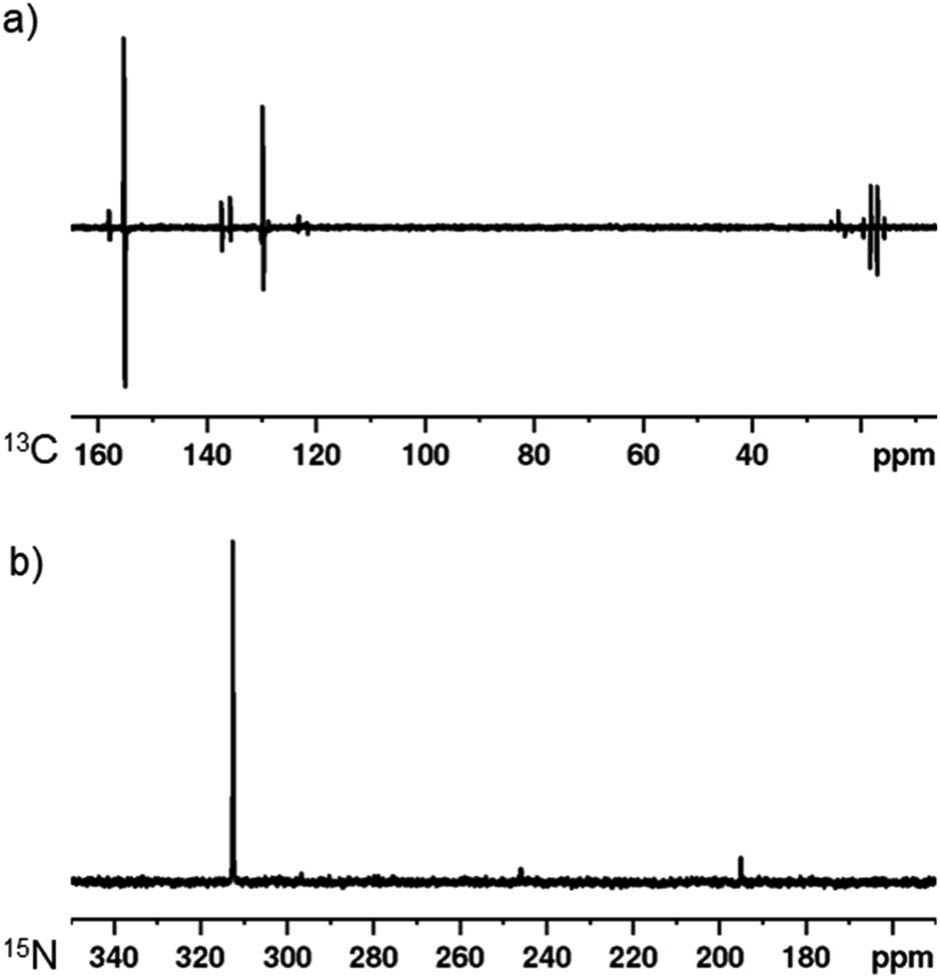
SABRE hyperpolarisation using [IrCl(COD)(1,3-bis(4-*tert*-butyl-2,6-dimethylphenyl)imidazole-2-ylidine)] (5 mM), DPSO (20 mM) and 2,5-lutidine (20 mM) in dichloromethane-*d*_2_ with 3 bar *p*-H_2_ after polarisation transfer at 273 K. (a) SABRE hyperpolarised ^13^C NMR spectrum after polarisation transfer at 0.5 G. (b) SABRE hyperpolarised ^15^N NMR spectrum after polarisation transfer at −3.5 mG.

As a final demonstration of this co-ligand strategy, polarisation transfer to ^13^C and ^15^N nuclei was achieved. A sample containing [IrCl(COD)(1,3-bis(4-*tert*-butyl-2,6-dimethylphenyl)imidazole-2-ylidine)] (5 mM), DPSO (20 mM) and 2,5-lutidine (20 mM) in dichloromethane-*d*_2_ was exposed to 3 bar *p*-H_2_ at 273 K in a 0.5 G polarisation transfer field. The subsequent ^13^C NMR spectrum is displayed in Fig. 4 and shows a 1424 ± 204-fold enhancement of the C2 of 2,5-lutidine (*δ* 155.2). Additionally, strong polarisation is seen throughout the carbon resonances with even the remote methyl groups being visible. Similarly, when a ^15^N{^1^H}^3–6,42^ NMR spectrum is obtained after the same sample is exposed to *p*-H_2_ in a −3.5 mG field, a 5048 ± 201-fold enhancement is seen at 9.4 T for the ^15^N resonance of 2,5-lutidine at *δ* 312.5.

## Conclusions

In summary, we present results that demonstrate how a co-ligand strategy can be used to successfully hyperpolarise weakly binding substrates using the signal amplification by reversible exchange methodology. Each of the substrates have functional groups in their *ortho* position that hinder their binding ability. Signal enhancements up to 1442 ± 84-fold are achieved for 2,5-lutidine. This is all the more remarkable as such substrates have previously been shown to be inaccessible to the SABRE technique when the commonly used [IrCl(COD)(IMes)] pre-catalyst is employed. Typically, this is due to the inability to form an active SABRE catalyst of type [Ir(H)_2_(IMes)(sub)_3_]Cl due to steric congestion around the metal centre.

Our strategy hinges on the use of a sulfoxide co-ligand to allow the formation of an active SABRE catalyst of the type [IrCl(H)_2_(NHC)(sulfoxide)(substrate)]. When [IrCl(COD)(IMes)] is exposed to H_2_ in the presence of dimethylsulfoxide-*d*_6_ (DMSO-*d*_6_) and 2,5-luditine a 1 : 2 mixture of [IrCl(H)_2_(IMes)(DMSO-*d*_6_)(2,5-lutidine)] and [IrCl(H)_2_(IMes)(DMSO-*d*_6_)_2_] were formed. When the sample was exposed to *p*-H_2_ in a 70 G polarisation transfer field polarisation transfer to 2,5-lutidine was observed. However, the presence of [IrCl(H)_2_(IMes)(DMSO-*d*_6_)_2_] in solution acts to rapidly consume *p*-H_2_ which is evidenced by broadening of the resonances for its hydride ligands and free H_2_ in the ^1^H NMR spectrum. We found that increasing the size of the sulfoxide co-ligand inhibits the formation of the bis-sulfoxide complex. Therefore, when diphenylsulfoxide (DPSO) is utilised no evidence of the corresponding [IrCl(H)_2_(IMes)(DPSO)_2_] complex is observed and an increased signal enhancement for 2,5-lutidine was quantified.

The effect of the *N*-heterocyclic carbene (NHC) was also probed and a *tert*-butyl derived catalyst, [IrCl(COD)(1,3-bis(4-*tert*-butyl-2,6-dimethylphenyl)imidazole-2-ylidine)],^14^ was shown to yield a complex which gave the best SABRE polarisation transfer to 2,5-lutidine. Further improvements were also discovered by cooling the sample to 273 K and a signal enhancement of 1442 ± 84-fold was achieved for 2,5-lutidine at 9.4 T after SABRE transfer at 70 G. The origin of this effect was determined to be due to the ameliorating the lifetime of the active catalyst. The rate of 2,5-lutidine loss at 273 K was determined to be 4.33 ± 0.02 s^−1^ which is extremely close to the predicted optimum of *ca.* 4.5 s^−1^.^36^ Additionally, SABRE hyperpolarised ^13^C and ^15^N NMR spectra were also recorded and signal enhancements of up to 1424 ± 204-fold and 5048 ± 201-fold were quantified respectively.

The scope of this method has been proven by expansion to 11 other substrates. The method is easy to implement as the standard SABRE pre-catalysts simply need to be mixed with the sulfoxide and substrate. It is noteworthy that the highest reported SABRE polarisation levels of 2-picoline, which has previously be reported for use as *in vivo* pH sensors,^43,44^ is given under the conditions reported here.

The methodology presented here therefore greatly expands the range of substrates amenable to the SABRE and is expected to open up new applications for the technique in the future. For example, as signal to noise scales with the square root of the number of measurements, these hyperpolarisation levels reflect remarkable measurement time reductions. Many active pharmaceuticals contain nitrogen heterocycles with sterically hindered nitrogen centres such as those in the diabetes treatment Januvia,^45^ the cancer treatments Zytiga^46^ and Sprycel,^47^ the immunosuppressant Xeljanz^48^ and the gastrointestinal drug Nexium.^49^ The route we illustrate here demonstrates how the detection of key ^1^H, ^13^C or ^15^N resonances in such materials can be achieved relatively simply in moments and work is being undertaken to render the SABRE method compatible for *in vivo* use.^50–56^

## Author contributions

P. J. R. and S. B. D. devised and oversaw the research. P. J. R., J. G. and V. D. H. prepared and analysed SABRE experiments. R. O. J. performed and interpreted DFT calculations. P. J. R. and S. B. D. wrote the manuscript with input from all authors.

## Conflicts of interest

There are no conflicts to declare.

## Supplementary Material

SC-012-D0SC06907H-s001
