# Supplementary material for: Hyperpolarisation of weakly binding N-heterocycles using signal amplification by reversible exchange
Source: Chem Sci. 2021 Mar 23;12(16):5910–7. doi: 10.1039/d0sc06907h (PMC8179664; doi:10.1039/d0sc06907h)
Supplement: SC-012-D0SC06907H-s001 [file SC-012-D0SC06907H-s001.pdf]

Electronic Supplementary Material (ESI) for Chemical Science.
This journal is © The Royal Society of Chemistry 2021

Supporting Information

Hyperpolarisation of weakly binding N-heterocycles using Signal Amplification by Reversible Exchange

Peter J. Rayner,[a] Joseph Gillions, [a] Valentin D. Hannibal, [a] Richard O. John and Simon B. Duckett,[a]

[a] Centre for Hyperpolarisation in Magnetic Resonance, Department of Chemistry, University of York, Heslington, YO10 5DD

1 NMR polarisation transfer experiment data

1.1 SABRE polarisation transfer method

The polarisation transfer experiments that are reported were conducted in 5 mm NMR tubes that were equipped with a J. Young's tap. Samples for these polarisation transfer experiments were based on a 5 mM solution of [IrCl(COD)(NHC)], co-ligand and the indicated additional substrate at the specified loading in methanol-d_4 or dichloromethane-d_2 (0.6 mL). The samples were degassed by two freeze-pump-thaw cycles prior to the introduction of *para*hydrogen at a pressure of 3 bar. *Para*-hydrogen (p-H_2) was produced by passing hydrogen gas over a spin-exchange catalyst (Fe_2O_3) at 28 K and used for all hyperpolarization experiments. This method produces constant p-H_2 with ca. 98% purity.

The shake & drop method was employed for recording hyperpolarized SABRE NMR spectra.[1] Once filled with p-H_2, samples were shaken vigorously for 10 s in the specified fringe field of an NMR spectrometer before being rapidly transported into the magnet for subsequent interrogation by NMR spectroscopy.

1.2 Polarization factors

^1H signal enhancements were calculated according to equation 1 where, E = enhancement level, SI(pol) = signal of polarized sample, SI(unpol) = signal of unpolarized (reference) sample.

$$E = \frac{SI(pol)}{SI(unpol)} \qquad (1)$$

Experimentally, both spectra were recorded on the same sample using identical acquisition parameters, including the receiver gain. The raw integrals of the relevant resonances in the polarized and unpolarised spectra were then used to determine the enhancement levels. The quoted values reflect the signal strength gain (fold) per proton nucleus in the specified group. The reference sample was allowed to equilibrate within the NMR spectrometer for 1-2 minutes prior to acquisition.

1.3 NMR Spectrometer

Spectra were typically acquired on a 400 MHz Bruker, Avance III console using a 5 mm BBI probe which was tuned to ^1H. Resonances are referenced relative to the residual proton signal of the indicated deuterated solvent.

2 NMR spectra of Hyperpolarised Substrates

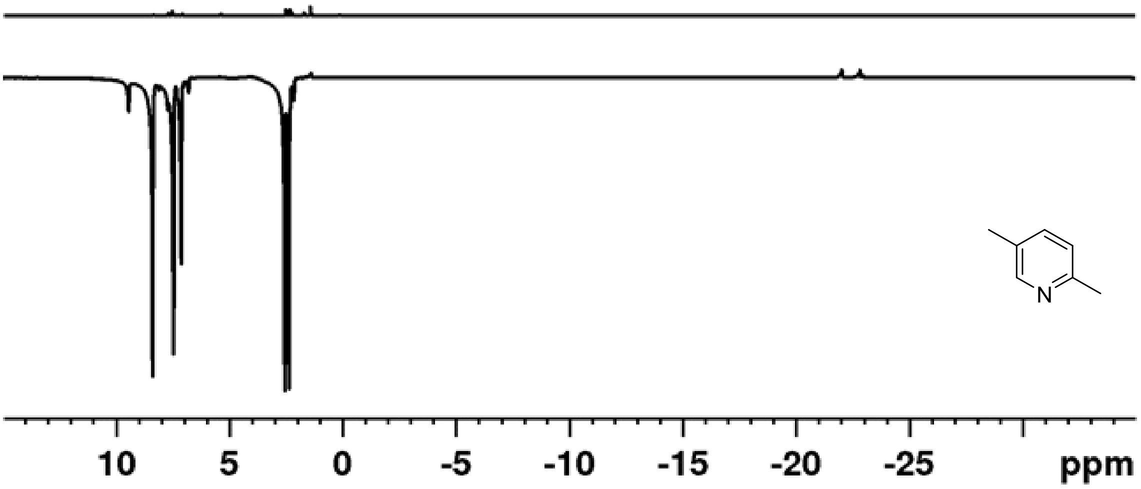

Figure S1: ^{1}H NMR spectra of 2,5-lutidine recorded under thermal (top) and SABRE (bottom) conditions. SABRE conditions: [IrCl(COD)(1,3-bis(4-tert-butyl-2,6-dimethylphenyl)imidazole-2-ylidine)] (5 mM), DPSO (20 mM) and 2,5-lutidine (20 mM) in dichloromethane-d_2 with 3 bar p-H_2 after transfer from a 70 G field.

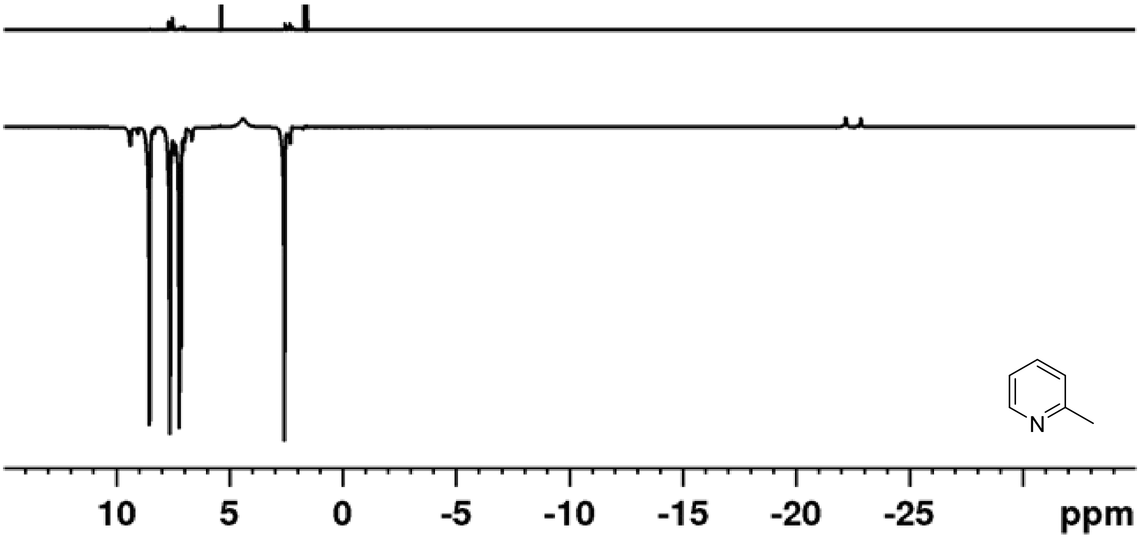

Figure S2: ^{1}H NMR spectra of 2-picoline recorded under thermal (top) and SABRE (bottom) conditions. SABRE conditions: [IrCl(COD)(1,3-bis(4-tert-butyl-2,6-dimethylphenyl)imidazole-2-ylidine)] (5 mM), DPSO (20 mM) and 2-picoline (20 mM) in dichloromethane-d_2 with 3 bar p-H_2 after transfer from a 70 G field.

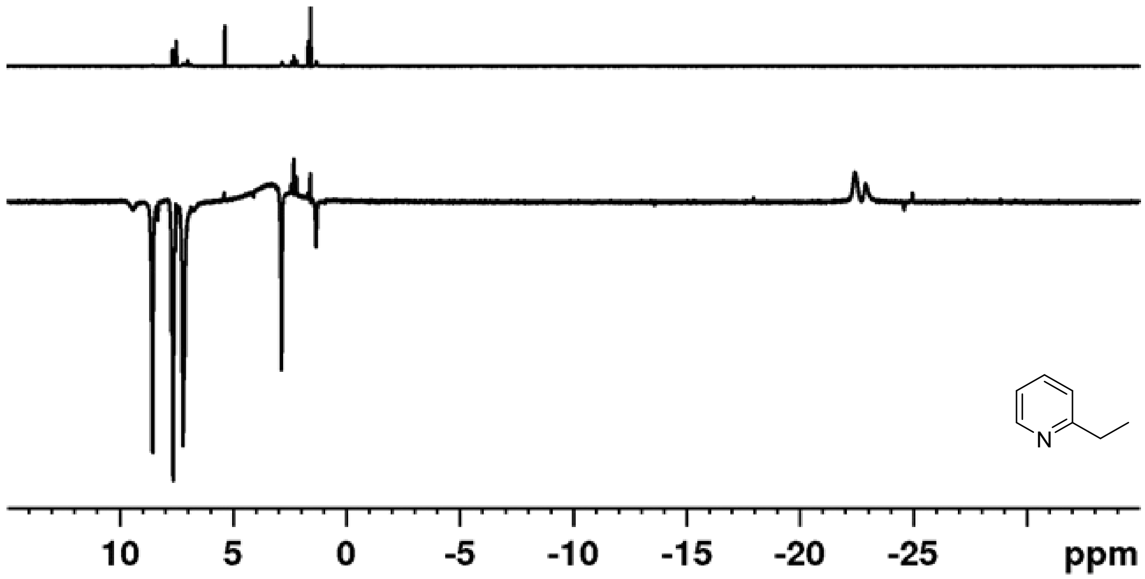

Figure S3: ^{1}H NMR spectra of 2-ethylpyridine recorded under thermal (top) and SABRE (bottom) conditions. SABRE conditions: [IrCl(COD)(1,3-bis(4-tert-butyl-2,6-dimethylphenyl)imidazole-2-ylidine)] (5 mM), DPSO (20 mM) and 2-ethylpyridine (20 mM) in dichloromethane-d_2 with 3 bar p-H_2 after transfer from a 70 G field.

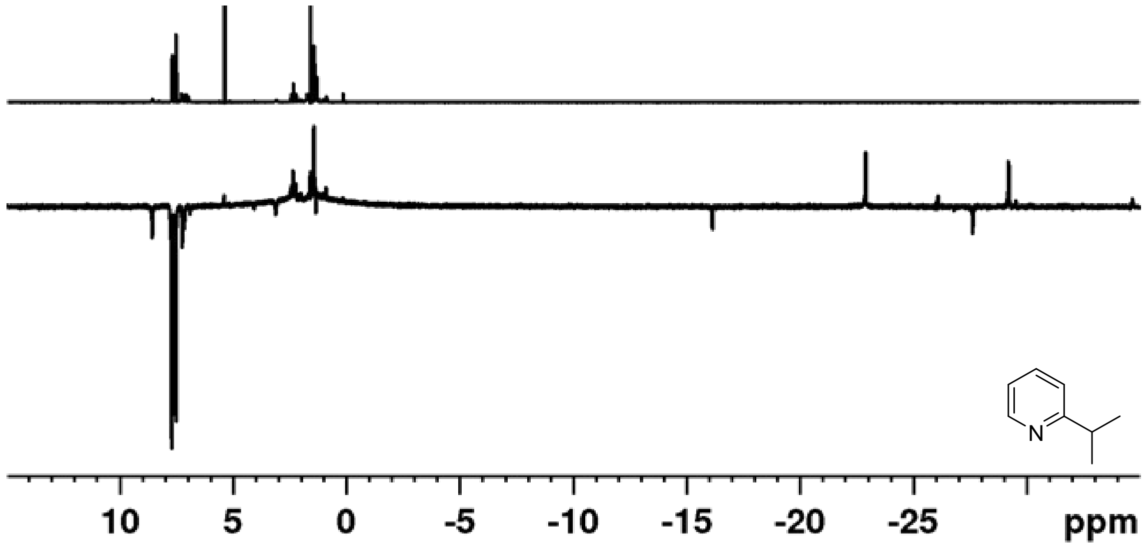

Figure S4: ^{1}H NMR spectra of 2-isopropylpyridine recorded under thermal (top) and SABRE (bottom) conditions. SABRE conditions: [IrCl(COD)(1,3-bis(4-tert-butyl-2,6-dimethylphenyl)imidazole-2-ylidine)] (5 mM), DPSO (20 mM) and 2-isopropylpyridine (20 mM) in dichloromethane-d_2 with 3 bar p-H_2 after transfer from a 70 G field.

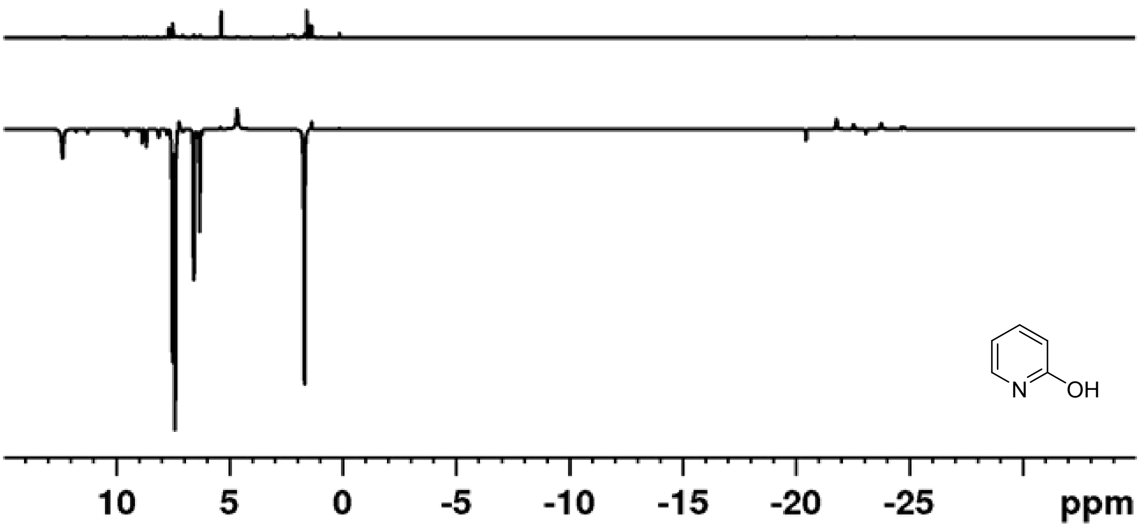

Figure S5: ^{1}H NMR spectra of 2-hydroxypyridine recorded under thermal (top) and SABRE (bottom) conditions. SABRE conditions: [IrCl(COD)(1,3-bis(4-tert-butyl-2,6-dimethylphenyl)imidazole-2-ylidine)] (5 mM), DPSO (20 mM) and 2-hydroxypyridine (20 mM) in dichloromethane-d_2 with 3 bar p-H_2 after transfer from a 70 G field.

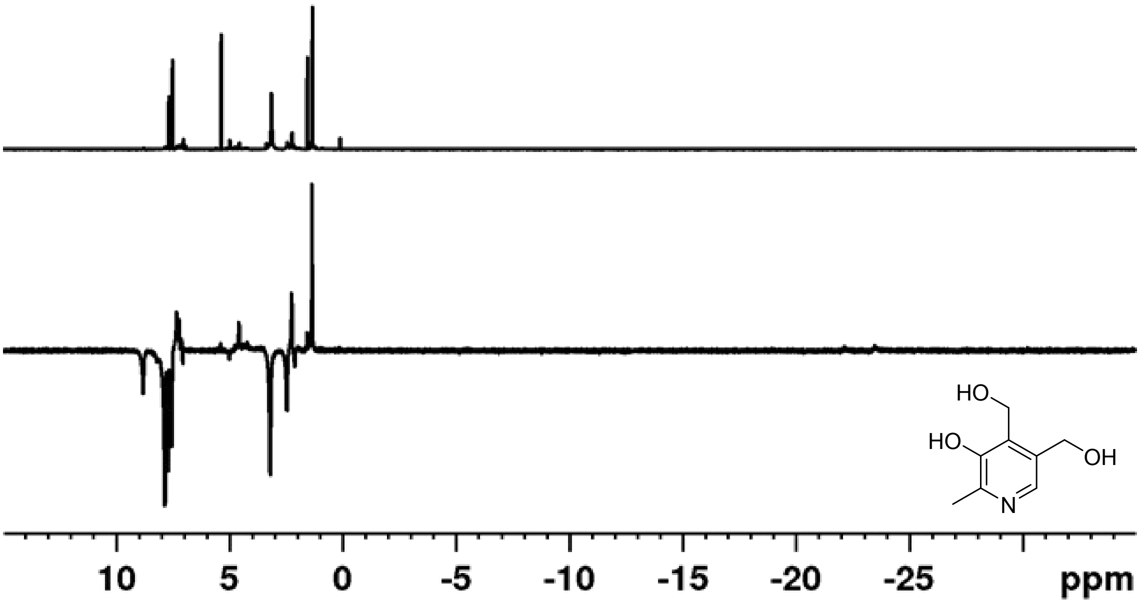

Figure S6: ^{1}H NMR spectra of pyridoxine recorded under thermal (top) and SABRE (bottom) conditions. SABRE conditions: [IrCl(COD)(1,3-bis(4-tert-butyl-2,6-dimethylphenyl)imidazole-2-ylidine)] (5 mM), DPSO (20 mM) and pyridoxine (20 mM) in dichloromethane-d_2 with 3 bar p-H_2 after transfer from a 70 G field.

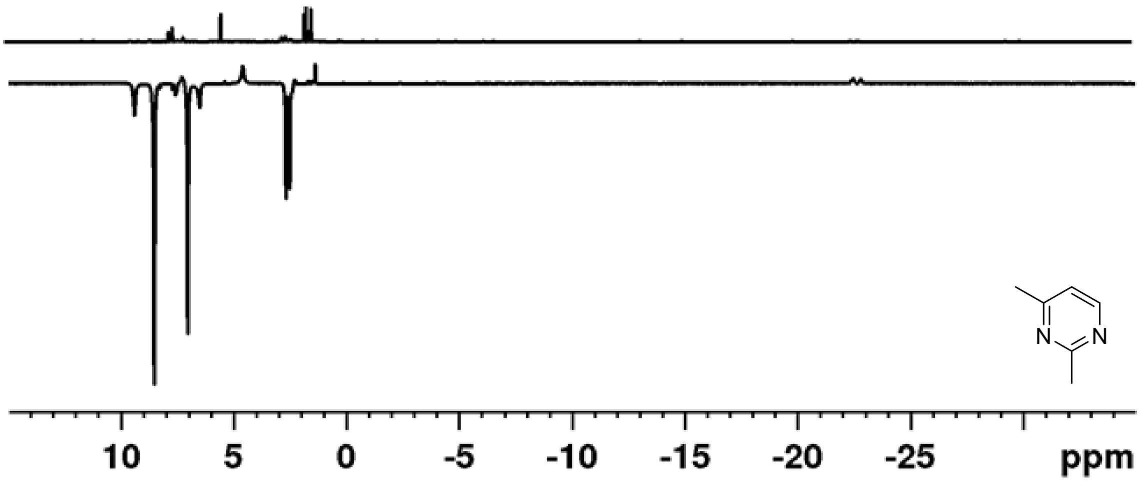

Figure S7: ^{1}H NMR spectra of 2,4-dimethylpyrimidine recorded under thermal (top) and SABRE (bottom) conditions. SABRE conditions: [IrCl(COD)(1,3-bis(4-tert-butyl-2,6-dimethylphenyl)imidazole-2-ylidine)] (5 mM), DPSO (20 mM) and 2,4-dimethylpyrimidine (20 mM) in dichloromethane-d_2 with 3 bar p-H_2 after transfer from a 70 G field.

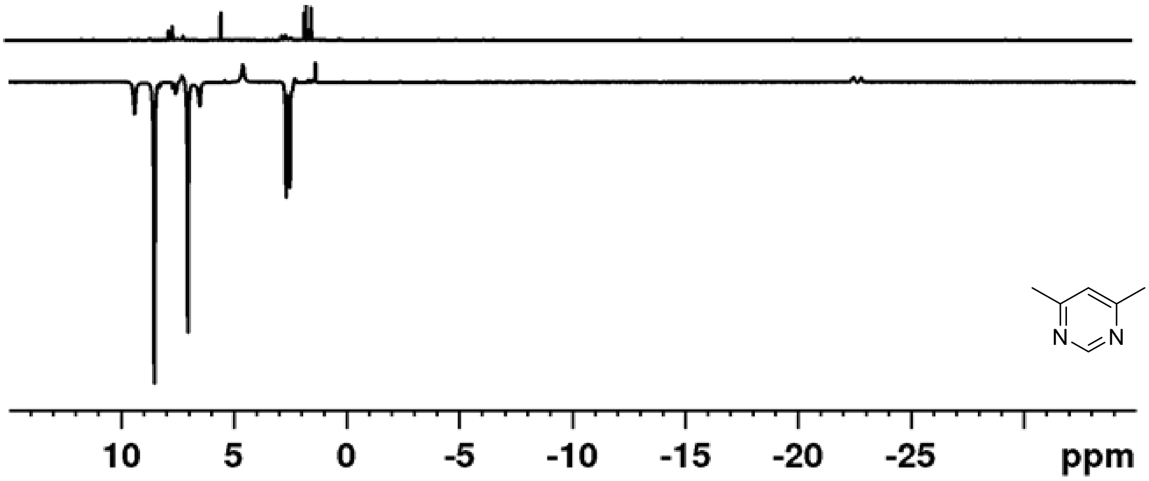

Figure S8: ^{1}H NMR spectra of 4,6-dimethylpyrimidine recorded under thermal (top) and SABRE (bottom) conditions. SABRE conditions: [IrCl(COD)(1,3-bis(4-tert-butyl-2,6-dimethylphenyl)imidazole-2-ylidine)] (5 mM), DPSO (20 mM) and 4,6-dimethylpyrimidine (20 mM) in dichloromethane-d_2 with 3 bar p-H_2 after transfer from a 70 G field.

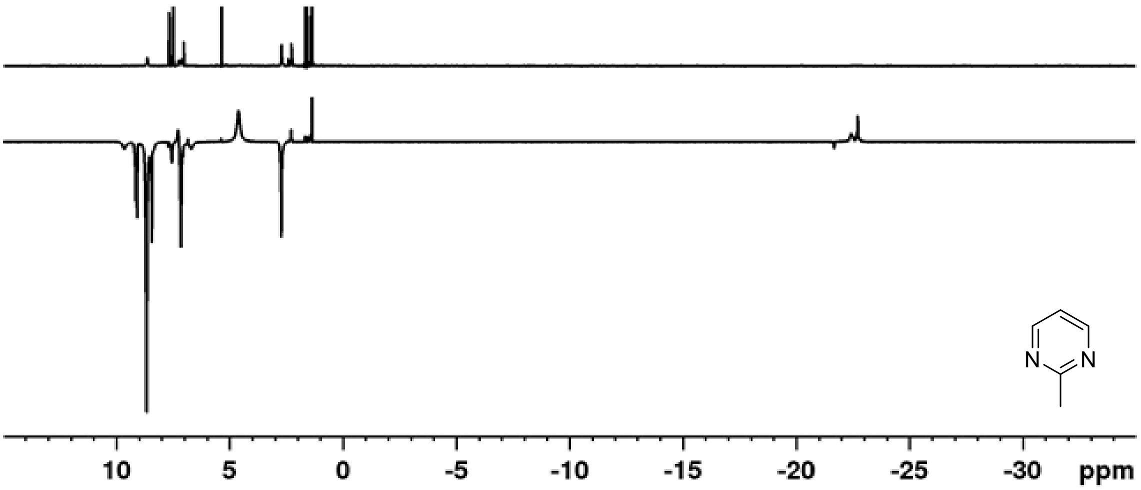

Figure S9: ^{1}H NMR spectra of 4-methylpyrimidine recorded under thermal (top) and SABRE (bottom) conditions. SABRE conditions: [IrCl(COD)(1,3-bis(4-tert-butyl-2,6-dimethylphenyl)imidazole-2-ylidine)] (5 mM), DPSO (20 mM) and 2-methylpyrimidine (20 mM) in dichloromethane-d_2 with 3 bar p-H_2 after transfer from a 70 G field.

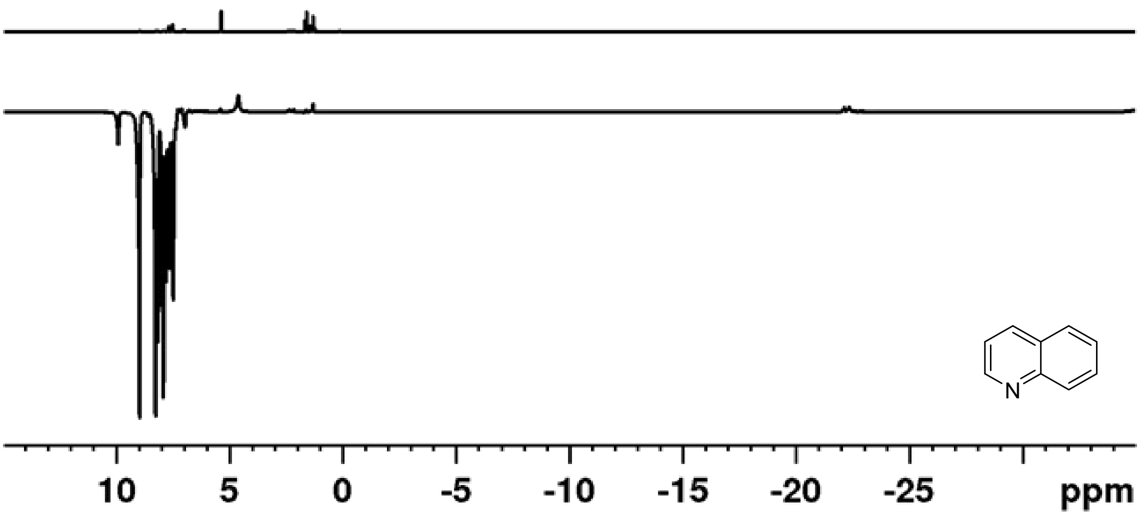

Figure S9: ^{1}H NMR spectra of quinoline recorded under thermal (top) and SABRE (bottom) conditions. SABRE conditions: [IrCl(COD)(1,3-bis(4-tert-butyl-2,6-dimethylphenyl)imidazole-2-ylidine)] (5 mM), DPSO (20 mM) and quinoline (20 mM) in dichloromethane-d_2 with 3 bar p-H_2 after transfer from a 70 G field.

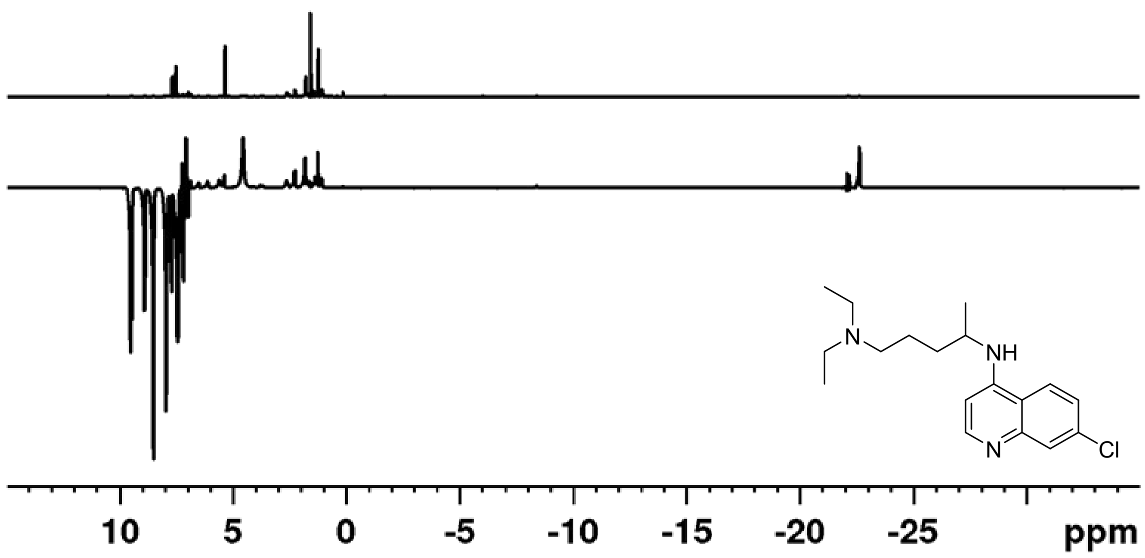

Figure S10: ^{1}H NMR spectra of chloroquine recorded under thermal (top) and SABRE (bottom) conditions. SABRE conditions: [IrCl(COD)(1,3-bis(4-tert-butyl-2,6-dimethylphenyl)imidazole-2-ylidine)] (5 mM), DPSO (20 mM) and chloroquine (20 mM) in dichloromethane-d_2 with 3 bar p-H_2 after transfer from a 70 G field.

3 Effect of Variation in DPSO Concentration

The effect of DPSO concentration on the 1H NMR signal enhancements of 2,5-lutidine was investigated. A series of NMR tubes containing incrementally increasing equivalents (0 – 10 eq.) of DPSO were prepared that contained a fixed concentration of [IrCl(COD)(IMes)] (5 mm), 2,5-lutidine (4 eq.) in dichlormethane-d_2 (0.6 mL). Each sample was shaken under 3 bar p-H_2 at 70 G and rapidly transferred into the NMR spectrometer for interrogation at 9.4 T. The resulting signal enhancements are displayed in Figure S11.

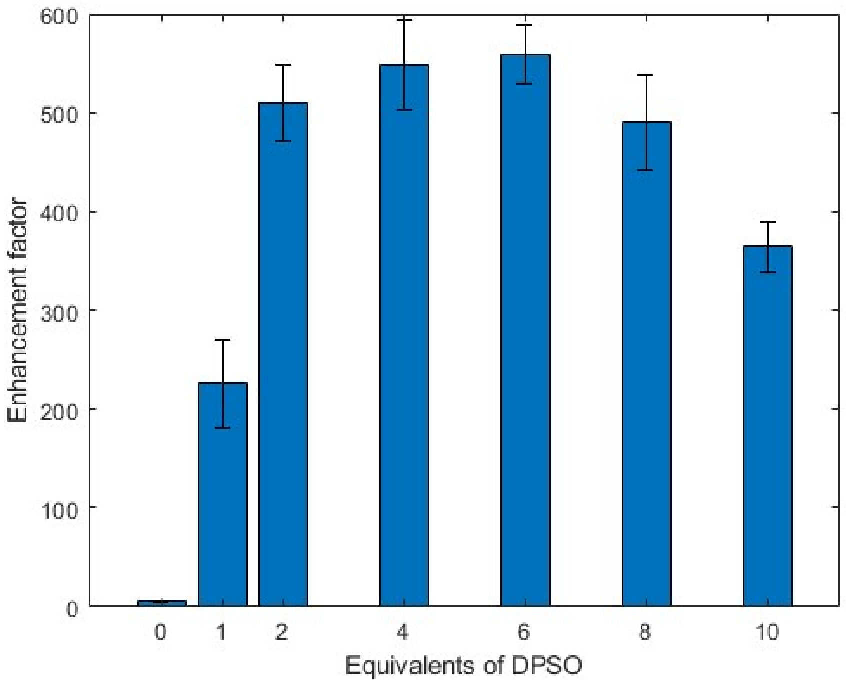

Figure S11: ^{1}H NMR signal enhancments as a function of DPSO equivalents relative to fixed concentration of [IrCl(COD)(IMes)] (5 mM), 2,5-lutidine (4 eq.) in dichloromethane-d_2.

As expected, when no DPSO is added there is no detectable signal enhancement and thus proving that DPSO is needed as a co-ligand in this process. The highest signal enhancements are obtained with between 2 and 6 equivalents of DPSO. This is consistent with an active catalyst of type [IrCl(H)$_2$(IMes)(DPSO)(2,5-lutidine)] being the dominant species in solution. When the DPSO concentration is increased above this level, the signal enhancement begins to decrease which could be due to competitive binding with 2,5-lutidine that would reduce the active catalyst concentration in solution.

4 Effect of catalyst counterion on SABRE signal enhancements

A series of catalysts that contained chloride, bromide, iodide or boron trifluoride counterions were synthesised and investigated for the effect on the SABRE hyperpolarization of 2,5-lutidine. Solutions containing each of the catalysts (5 mM), 2,5-lutidine (20 mM), DPSO (20 mM) in dichloromethane-d_2 were exposed to p-H_2 (3 bar) in a 70 G polarisation transfer field before interrogation by ^{1}H NMR spectroscopy. The resulting ^{1}H NMR signal gains are shown in Table S1.

Table S1: Effect of catalysts counterion of the ^{1}H NMR signal enhancements of 2,5-lutidine

**Pre-catalyst**	**^{1}H NMR Signal Enhancement for the ortho-proton of 2,5-lutidine**
[IrCl(COD)(IMes)]	723 ± 38
[IrBr(COD)(IMes)]	579 ± 18
[IrI(COD)(IMes)]	171 ± 14
[Ir(COD)(IMes)(2,5-lutidine)]BF_4	19 ± 8

[IrCl(COD)(IMes)] gave the largest signal enhancements for the ortho proton of 2,5-lutidine. The signal enhancements decrease when either the bromide or iodide precatalysts are employed. When the BF_4 counterion is utilised, the signal enhancements drop significantly to just 19 ± 8-fold and therefore highlights the significance of a chelating ligand in the SABRE polarisation transfer mechanism for sterically hindered substrates. This is consistent with DFT calculations (see section 7) that show that the formation of [Ir(H)$_2$(IMes)(2,5-lutidine)(DPSO)$_2$] or [Ir(H)$_2$(IMes)(2,5-lutidine)$_2$(DPSO)] is disfavored when compared to [IrCl(H)$_2$(IMes)(2,5-lutidine)(DPSO)]

4.2 Effect of chloride concentration on SABRE signal enhancements

The concentration of chloride on the ^{1}H NMR signal enhancement of 2,5-lutidine was also investigated. NBu_4Cl was used a chloride donor by adding increasing concentrations to a series of NMR tubes containing [IrCl(COD)(IMes)] (5 mM), 2,5-lutidine (4 eq.), DPSO (4 eq.) in dichloromethane-d_2. The signal enhancement was shown to be independent of chloride concentration as shown in Figure S12. Similar data was recorded when NaCl was used as the chloride donor.

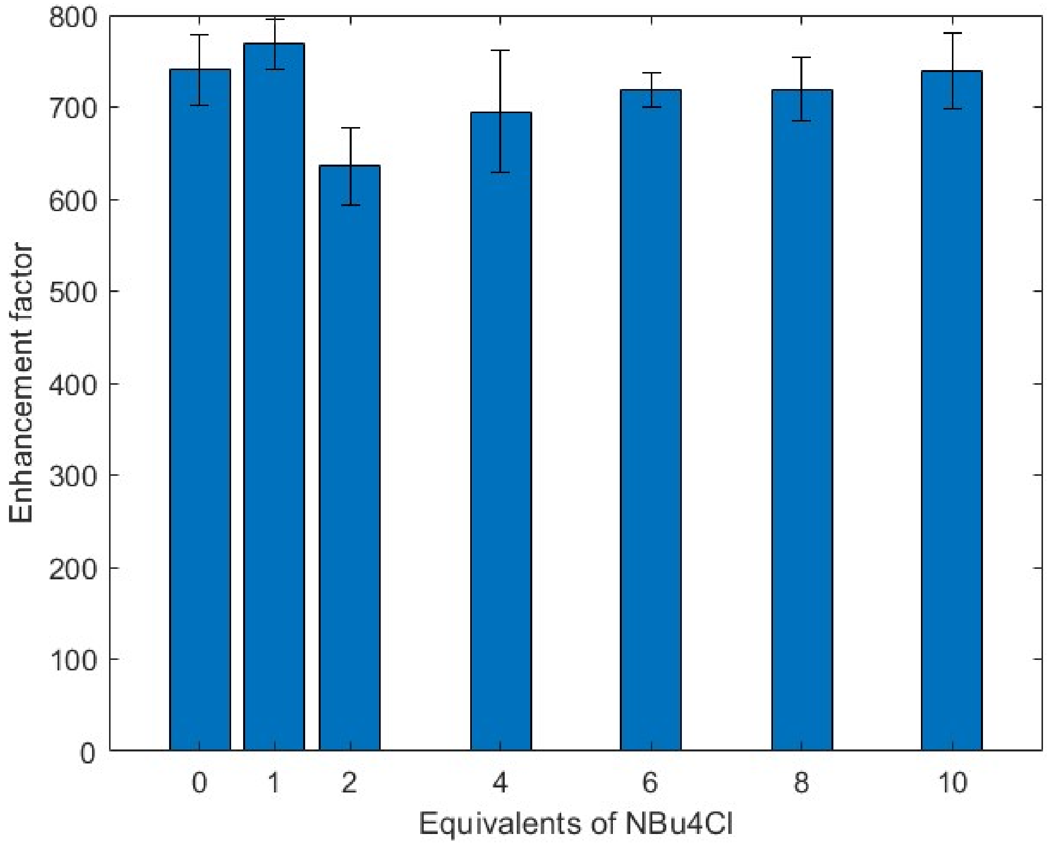

Figure S12: ¹H NMR signal enhancements as a function of NBu_4Cl equivalents relative to fixed concentration of [IrCl(COD)(IMes)] (5 mM), 2,5-lutidine (4 eq.) and DPSO (4 eq.) in dichloromethane-*d*$_2$.

5 Effect of variation to the N-heterocyclic carbene ligand

A series of N-heterocyclic carbene (NHC) ligands were selected based on their Tolmann Electronic Parameters (TEP) and buried volumes (%V_{bur}) to investigate their effect on the SABRE hyperpolarisation of 2,5-lutidine. Their synthetic procedures have been reported previously.[2] The signal enhancements for the ortho proton of 2,5-lutidine after SABRE transfer (using [IrCl(COD)(NHC)] (5 mM), 2,5-lutidine (4 eq.), DPSO (4 eq.) in dichloromethane-d_2 (0.6 mL) under 3 bar at 70 G) are reported in Figure S14.

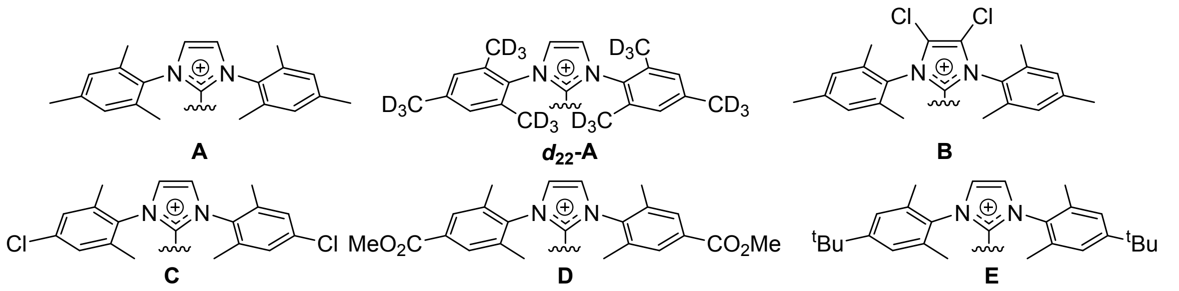

Figure S13: Structures of N-heterocyclic carbene (NHC) ligands for the precatalysts of the form [IrCl(COD)(NHC)].

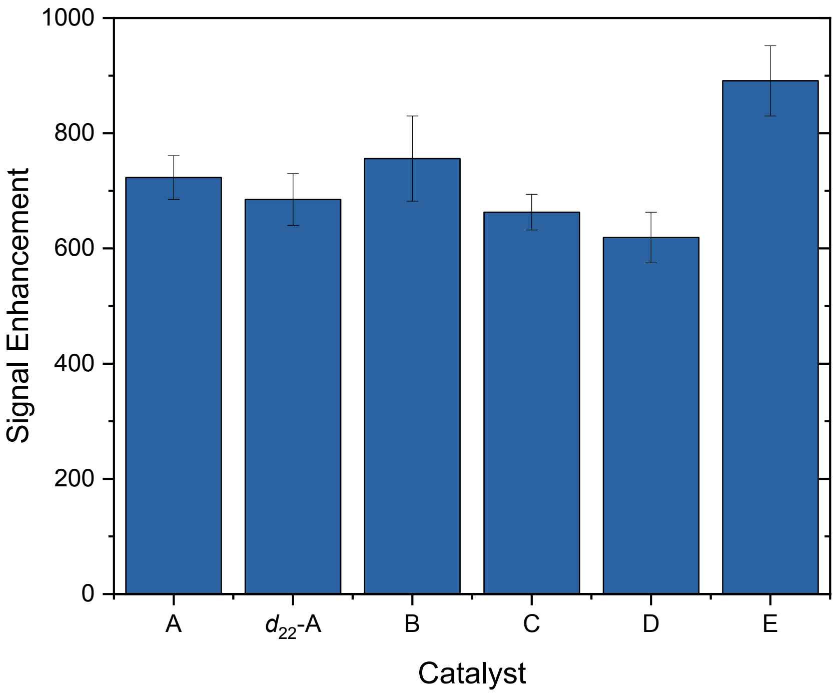

Figure S14: ^{1}H NMR signal enhancements when different [IrCl(COD)(NHC)] (5 mM) catalysts are used in the presence of 2,5-lutidine (4 eq.) and DPSO (4 eq.) in dichloromethane-d_2.

Figure S14 shows that catalyst **E** which bears *tert*-butyl groups in the para position of the aryl rings gives significantly higher signal enhancements that the [IrCl(COD)(IMes)] catalyst. The other catalysts give comparable or reduced signal enhancements to [IrCl(COD)(IMes)].

6 Synthetic Methods and Characterisation Data

6.1 Preparation of $[Ir(COD)(IMes)(2,5\text{-lutidine})]BF_4$

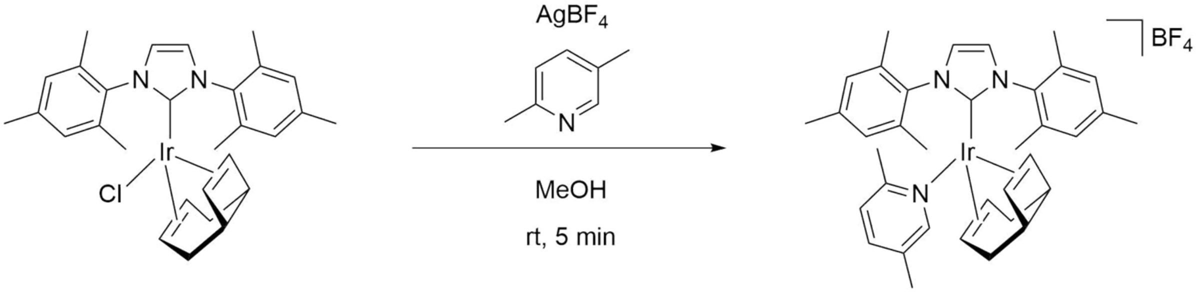

Figure S15: Synthesis of $[Ir(COD)(IMes)(2,5\text{-lutidine})]BF_4$

$AgBF_4$ (7.61 mg, 39.1 µmol, 1.00 eq) was added to a stirred solution of [IrCl(COD)(IMes)] (25.0 mg, 39.1 µmol, 1.00 eq) and 2,5-lutidine (4.50 µl, 39.1 µmol, 1.00 eq) in MeOH (5 ml). The solution was stirred at room temperature for 5 minutes and then the precipitate is removed by filtration. The solvent was removed under reduced pressure to give $[Ir(2,5\text{-lutidine})(COD)(IMes)]BF_4$ (28.6 mg, 35.9 µmol, 91.8 %), **^{1}H NMR**: δ [ppm] = 7.58 (d, *J* = 7.9 Hz, 1H), 7.35 (s, 2H), 7.30 (d, *J* = 7.9 Hz, 1H),7.24 (s, 2H), 7.11 (s, 2H), 6.98 (s, 1H), 4.33-4.30 (m, 1H), 3.72-3.69 (m, 1H), 3.46-3.41 (m, 1H), 2.83 (s, 3H), 2.72-2.67 (m, 1H), 2.18 (s, 6H), 2.08 (s, 3H), 2.08 (s, 6H), 2.07-2.01 (m, 2H), 1.90-1.86 (m, 2H), 1.86 (s, 6H), 1.74-1.69 (m, 2H), 1.59-1.53 (m, 2H); **^{13}C NMR**: δ [ppm] = 173.6 (Cq, 1C), 156.6 (Cq, 2C), 149.1 (CH, 1C), 140.0 (Cq, 2C), 138.1(CH, 1C), 135.9 (Cq, 1C), 135.7 (Cq, 2C), 135.6 (Cq, 2C), 132.7 (Cq, 1C), 129.4 (CH, 2C),129.1 (CH, 2 C), 126.3 (CH, 1 C), 125.4 (Cq, 1C), 85.1 (CH, 1C), 76.3 (CH, 1C), 67.1 (CH,1C), 58.1 (CH, 1C), 34.2 (CH_2, 1C), 31.0 (CH_2, 1C), 30.0 (CH_2, 1C), 26.6 (CH_2, 1C), 25.1(CH_3, 1C), 19.8 (CH_3, 2C), 17.4 (CH_3, 2C), 16.9 (CH_3, 1C), 16.7 (CH_3, 1C).

6.2 Identification of the Active Catalyst

Table S2: ^{1}H NMR signal assignments

^{1}H NMR data

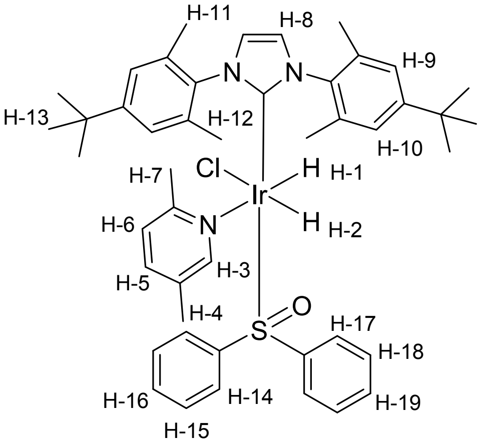

δ [ppm] (info)	Assignment
9.33 (s, 1 H)	H-3
7.62-7.60 (m, 2 H)	H-14 or H-17
7.46 (m, 2 H)	H-14 or H-17
7.31-7.29 (m, 3 H)	H-15/16 or H-18/19
7.17-7.16 (m, 3 H)	H-15/16 or H-18/19
7.13 (d, $^{3}J_{HH}$ = 8.0 Hz, 1 H)	H-5
7.09 (s, 2 H)	H-9 or H-10
7.04 (s, 2 H)	H-9 or H-10
7.00 (s, 2 H)	H-8
6.75 (d, $^{3}J_{HH}$ = 8.0 Hz, 1 H)	H-6
2.28 (s, 6 H)	H-11 or H-12
2.22 (s, 6 H)	H-11 or H-12
2.15 (s, 3 H)	H-4
2.12 (s, 3 H)	H-7
1.31 (s, 18 H)	H-13
−21.69 (d, $^{3}J_{HH}$ = 7.1 Hz, 1 H)	H-1
−22.94 (d, $^{3}J_{HH}$ = 7.1 Hz, 1 H)	H-2

Table S3: ^{15}N NMR signal assignments

^{15}N NMR data		
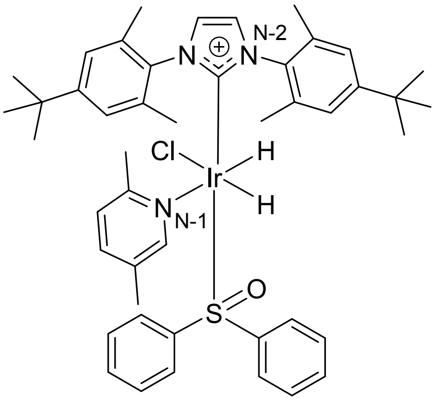	***δ* [ppm] (info)**	**Assignment**
	246.1 (s)	N-1
	195.2	N-2

Table S4: ^{13}C NMR signal assignments

^{13}C NMR data		
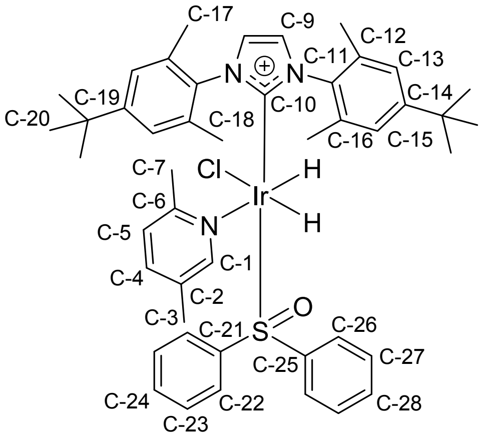	***δ* [ppm] (info)**	**Assignment**
	157.9	C-10
	153.9 (s, 1C)	C-1
	150.1	C-14
	146.7	C-10
	137.9	C-11
	136.7 (s, 1C)	C-4
	135.1	C-12 or C-16
	134.3	C-2
	131.2	C-21 or C-25
	129.9	C-12 or C-16
	129.5	C-21 or C-25
	129.0 (s, 1C)	C-6

	127.9 (s, 2C)	C-23 or C-27
	127.6 (s, 2C)	C-23 or C-27
	125.8 (s,)	C-22 or C-26
	125.5	C-24 or C-28
	125.4 (s, 2C)	C-22 or C-26
	125.1 (s, 2C)	C-13 or C-15
	124.8	C-24 or C-28
	124.3 (s, 2C)	C-13 or C-15
	123.4 (1C)	C-5
	122.7 (2C)	C-9
	34.4 (2C)	C-19
	31.2 (6C)	C-20
	28.6 (1C)	C-7
	19.6 (1C)	C-3
	18.0 (2C)	C-17 or C-18

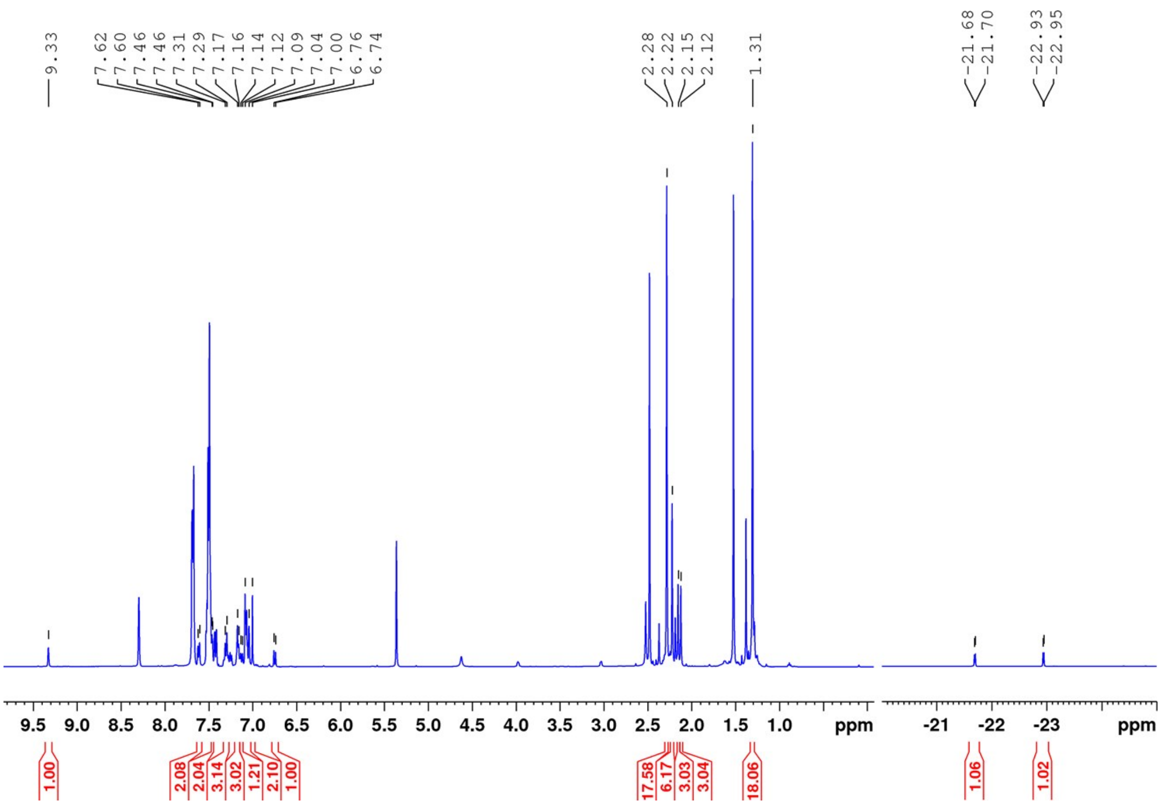

Figure S16: ^{1}H NMR spectrum (DCM-d_2, 400.1 MHz, 243 K).

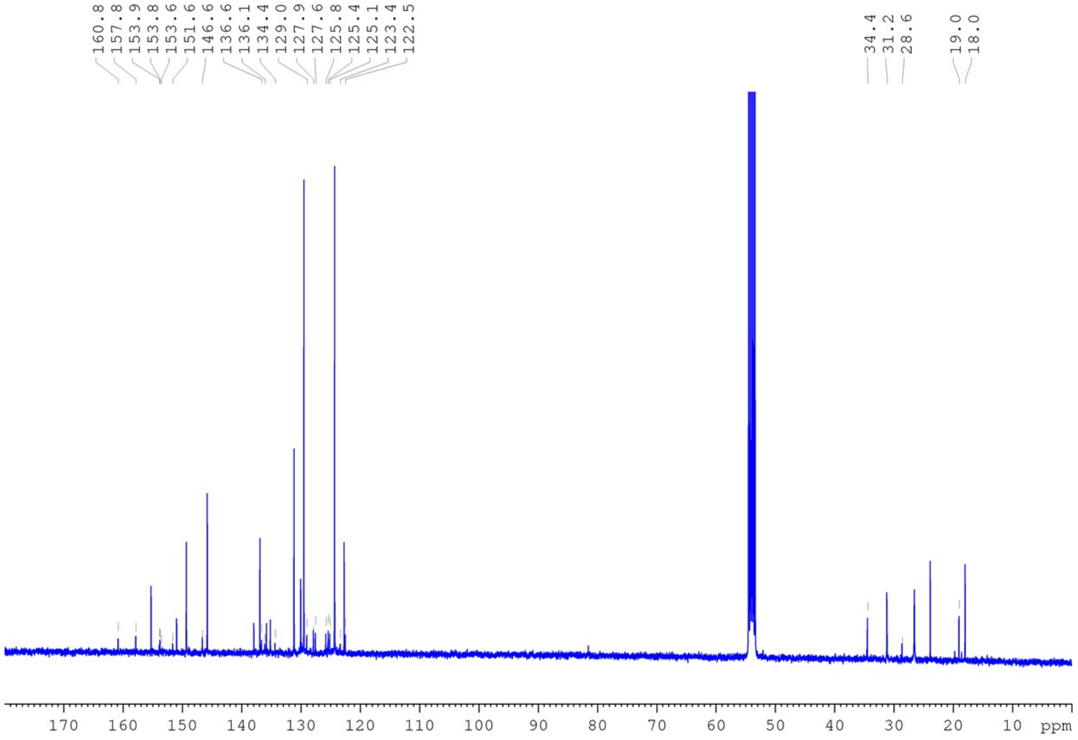

Figure S17: ^{13}C NMR spectrum (DCM-d_2, 100.6 MHz, 243 K).

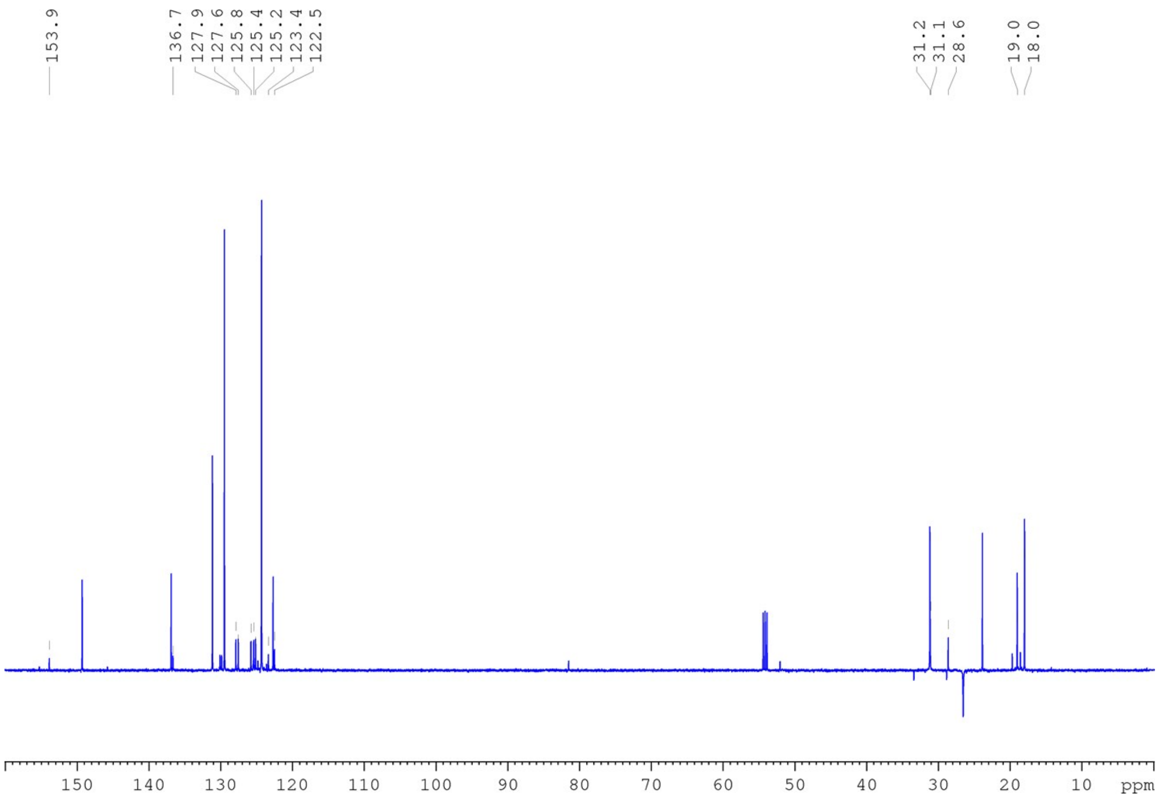

Figure S18: ^{13}C DEPT-135 NMR spectrum (DCM-d_2, 400.1/100.6 MHz, 243 K).

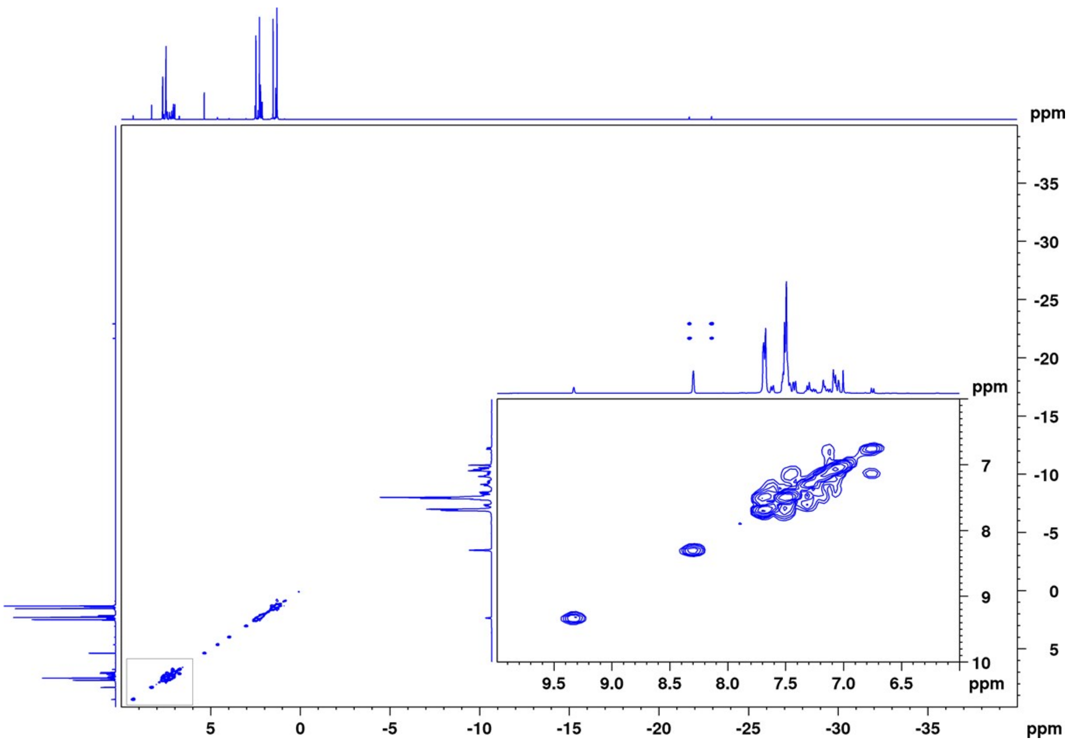

Figure S19: ^{1}H-^{1}H COSY NMR spectrum (DCM-d_2, 400.1 MHz, 243 K):

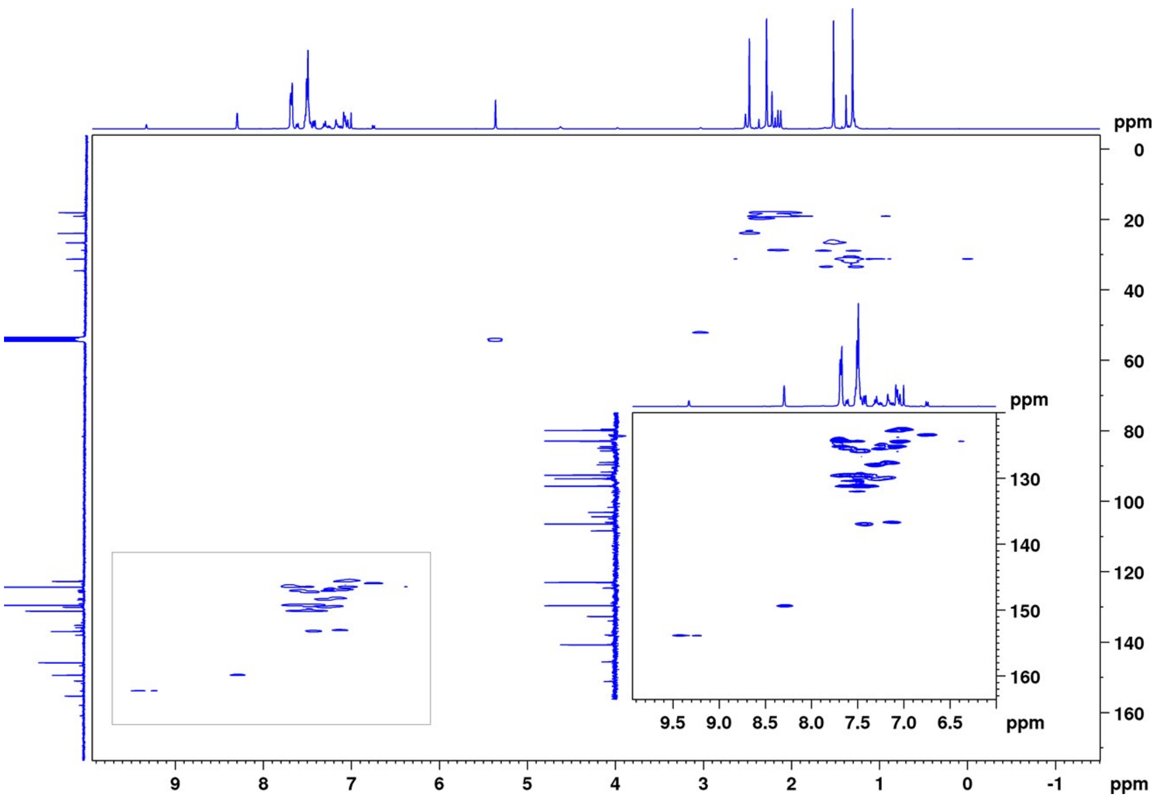

Figure S20: ^{1}H-^{13}C HSQC NMR spectrum (DCM-d_2, 400.1/100.6 MHz, 243K):

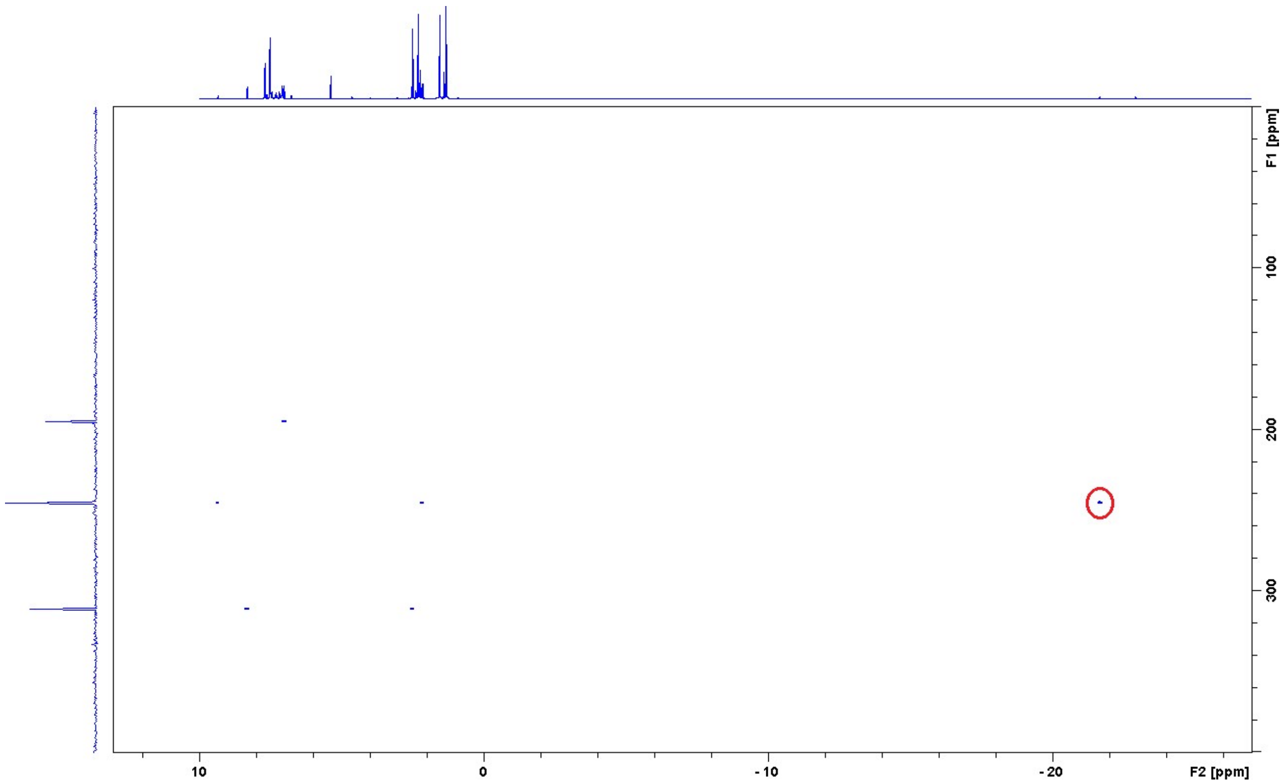

Figure S21: ^{1}H-^{15}N HMQC NMR spectrum (DCM-d_2, 400.1/40.5 MHz, 243K):

7 Density Functional Theory

All calculations were performed using the GAUSSIAN 09 series of programs[3] using the PBE0 functional[4-6]. An effective core potential and its associated double-ζ LANL2TZ basis set[7, 8] with additional f polarisation functions[9] was used for the iridium atoms. All remaining atoms were assigned to the def2-SVP basis sets[10, 11]. The calculations here employed the full ligand set to correctly account for steric interactions. The structures of the reactants, intermediates, transition states, and products were fully optimized without any symmetry restriction. Frequency calculations were performed on all optimized structures at the same level of theory to characterize the stationary points and the transitions states, as well as for the calculation of zero-point energies (ZPE), enthalpies (H), entropies (S), and Gibbs energies (G) at 298.15 K. Single point calculations with the PBE0 functional along with the same LANL2TZ basis set for iridium were then used with the larger def2-TZVPP basis sets[10, 11] to allow more accurate energies to be obtained. All energetics were counterpoise corrected for the effects of basis set superposition error[12, 13]. Solvent effects were included in all calculations with the IEFPCM model[14-16].

Model	SCF Energy / Hartree	Thermal correction to Gibbs Free Energy / Hartree
$[IrCl(H)_2(IMes)(DMSO)(2,5\text{-lutidine})]$	-2369.171201	0.572499
$[IrCl(H)_2(IMes)(DMSO)_2]$	-2595.498575	0.513643
$[Ir(H)_2(IMes)(DMSO)_2(2,5\text{-lutidine})]$	-2461.900895	0.654042
$[IrCl(H)_2(IMes)(2,5\text{-lutidine})_3]$	-2009.238391	0.773023
$[IrCl(H)_2(IMes)(DPSO)(2,5\text{-lutidine})]$	-2752.305957	0.669679
$[IrCl(H)_2(IMes)(DPSO)_2]$	-3361.777344	0.80038
$[IrCl(H)_2(IMes)(DPSO)(quinoline)]$	-2827.256613	0.662877
Cl^-	-460.231442	-0.015023
2,5-lutidine	-326.6506531	0.110582
DMSO	-552.9812427	0.051134
DPSO	-936.1266662	0.148222
Quinoline	-401.6000017	0.105849

8 References

1. R. W. Adams, J. A. Aguilar, K. D. Atkinson, M. J. Cowley, P. I. Elliott, S. B. Duckett, G. G. Green, I. G. Khazal, J. López-Serrano and D. C. Williamson, *Science*, 2009, **323**, 1708-1711.
2. P. J. Rayner, P. Norcott, K. M. Appleby, W. Iali, R. O. John, S. J. Hart, A. C. Whitwood and S. B. Duckett, *Nat. Commun.*, 2018, **9**, 4251.
3. G. W. T. M. J. Frisch, H. B. Schlegel, G. E. Scuseria, , J. R. C. M. A. Robb, G. Scalmani, V. Barone, B. Mennucci, , H. N. G. A. Petersson, M. Caricato, X. Li, H. P. Hratchian, , J. B. A. F. Izmaylov, G. Zheng, J. L. Sonnenberg, M. Hada, , K. T. M. Ehara, R. Fukuda, J. Hasegawa, M. Ishida, T. Nakajima, , O. K. Y. Honda, H. Nakai, T. Vreven, J. A. Montgomery, Jr., , F. O. J. E. Peralta, M. Bearpark, J. J. Heyd, E. Brothers, , V. N. S. K. N. Kudin, T. Keith, R. Kobayashi, J. Normand, , A. R. K. Raghavachari, J. C. Burant, S. S. Iyengar, J. Tomasi, , N. R. M. Cossi, J. M. Millam, M. Klene, J. E. Knox, J. B. Cross, , C. A. V. Bakken, J. Jaramillo, R. Gomperts, R. E. Stratmann, , A. J. A. O. Yazyev, R. Cammi, C. Pomelli, J. W. Ochterski, , K. M. R. L. Martin, V. G. Zakrzewski, G. A. Voth, , J. J. D. P. Salvador, S. Dapprich, A. D. Daniels, , J. B. F. O. Farkas, J. V. Ortiz, J. Cioslowski, and and D. J. Fox, *Gaussian 09, Revision D.01*, 2013, Wallingford CT.
4. J. P. Perdew, K. Burke and M. Ernzerhof, *Phys. Rev. Lett.*, 1996, **77**, 3865-3868.
5. J. P. Perdew, K. Burke and M. Ernzerhof, *Phys. Rev. Lett.*, 1997, **78**, 1396.
6. C. Adamo and V. Barone, *J. Chem. Phys.*, 1999, **110**, 6158-6170.
7. P. J. Hay and W. Wadt, *J. Chem. Phys.*, 1985, **82**, 299-310.
8. L. E. Roy, P. J. Hay and R. L. Martin, *J. Chem. Theory Comput.*, 2008, **4**, 1029-1031.
9. A. W. Ehlers, M. Bohme, S. Dapprich, A. Gobbi, A. Hollwarth, V. Jonas, K. F. Kohler, R. Stegmann, A. Veldkamp and G. Frenking, *Chem. Phys. Lett.*, 1993, **208**, 111-114.
10. A. Schafer, H. Horn and R. Ahlrichs, *Journal of Chemical Physics*, 1992, **97**, 2571-2577.
11. F. Weigend and R. Ahlrichs, *Physical Chemistry Chemical Physics*, 2005, **7**, 3297-3305.
12. S. F. Boys and F. Bernadi, *Mol. Phys.*, 1970, **19**, 553-553.
13. S. Simon, M. Duran and J. J. Dannenberg, *J. Chem. Phys.*, 1996, **105**, 11024-11031.
14. E. Cances, B. Mennucci and J. Tomasi, *J. Chem. Phys.*, 1997, **107**, 3032-3041.
15. B. Mennucci, E. Cances and J. Tomasi, *J. Phys. Chem. B*, 1997, **101**, 10506-10517.
16. E. Cances and B. Mennucci, *J. Math. Chem.*, 1998, **23**, 309-326.
